# The impact of AI suggestions on radiologists’ decisions: a pilot study of explainability and attitudinal priming interventions in mammography examination

**DOI:** 10.1038/s41598-023-36435-3

**Published:** 2023-06-07

**Authors:** Mohammad H. Rezazade Mehrizi, Ferdinand Mol, Marcel Peter, Erik Ranschaert, Daniel Pinto Dos Santos, Ramin Shahidi, Mansoor Fatehi, Thomas Dratsch

**Affiliations:** 1 Vrije Universiteit Amsterdam, Amsterdam, The Netherlands; 2 Ghent University, Ghent, Belgium; 3 Institute of Diagnostic and Interventional Radiology, Faculty of Medicine and University Hospital Cologne, University of Cologne, Cologne, Germany; 4 Bushehr University of Medical Sciences, Bushehr, Iran; 5 National Brain Mapping Laboratory, Tehran, Iran

**Keywords:** Preclinical research, Mathematics and computing, Information technology, Human behaviour

## Abstract

Various studies have shown that medical professionals are prone to follow the incorrect suggestions offered by algorithms, especially when they have limited inputs to interrogate and interpret such suggestions and when they have an attitude of relying on them. We examine the effect of correct and incorrect algorithmic suggestions on the diagnosis performance of radiologists when (1) they have no, partial, and extensive informational inputs for explaining the suggestions (study 1) and (2) they are primed to hold a positive, negative, ambivalent, or neutral attitude towards AI (study 2). Our analysis of 2760 decisions made by 92 radiologists conducting 15 mammography examinations shows that radiologists’ diagnoses follow both incorrect and correct suggestions, despite variations in the explainability inputs and attitudinal priming interventions. We identify and explain various pathways through which radiologists navigate through the decision process and arrive at correct or incorrect decisions. Overall, the findings of both studies show the limited effect of using explainability inputs and attitudinal priming for overcoming the influence of (incorrect) algorithmic suggestions.

## Introduction

The introduction of data-driven algorithms in the domain of medical imaging is one of the leading areas of technological development. A distinguishing characteristic of new algorithms is their black-box character: the complexity of understanding the relations between inputs and outputs^1^. Especially in the medical context, this has major implications for the development and deployment of these algorithms since medical decisions are high stakes and carry strict legal liabilities^2,3^.

Several studies have shown that (medical) professionals are prone to be impacted by the suggestions and inputs provided by various forms of algorithmic tools such as computer-aided detection (CAD) and different forms of artificial intelligence (AI). CAD, a precursor to more advanced AI-based systems, has been extensively studied for its effects on diagnostic accuracy and the potential for misuse. Despite their potential, various studies have found that CAD tools can decrease the specificity and sensitivity of radiologists’ decisions^4–7^. For instance, in examining mammograms, radiologists who were assisted by CAD were more likely to miss pathological findings when such indications were also missed in the CAD results^4^. The results of another study demonstrated that CAD improved sensitivity for relatively easy-to-detect cancers, but decreased sensitivity for more difficult cases^5^. This decrease in sensitivity for challenging cases was attributed to radiologists' increased reliance on CAD when they were uncertain of their own decisions^8^.

Furthermore, studies have shown that the potential for CAD misuse is heightened when used as a concurrent reader (immediate availability of CAD output) rather than a second reader. For instance, researchers discovered that a poorly performing CAD system used concurrently significantly diminished radiologists' diagnostic performance in mammogram reading^9,10^. Similarly, researchers found that employing CAD as a concurrent reader significantly reduced the reading time compared to radiologists working without CAD assistance^11^. Consequently, this reduction in thoroughness was associated with a diminished sensitivity in comparison to readings conducted without CAD support^11^.

Despite ample research on designing data-driven algorithms, there is relatively limited research on the interactions that emerge when medical professionals interact with the results offered by these algorithms^12,13^. This challenge is exacerbated in practice when medical professionals are presented with the “outcome suggestions” made by the algorithms (e.g., malignancy score of tissues), with limited informational inputs to interrogate and interpret how such suggestions are derived from the specific inputs.

Ideally, professionals engage in “reflective practices” through which they directly inspect medical images and informational inputs, and consider various medical scenarios, compared to the situation in which they do not have such suggestions^13^. However, in practice, forms of limited reflective engagement with AI suggestions have been documented by various studies. Here, two mechanisms of cognitive bias are likely to occur: (1) *over-reliance*, agreement with AI suggestions with no or limited reflective engagement and (2) *under-reliance*, disagreement with AI suggestions with no or limited reflective engagement^14^.

Over-reliance on algorithmic suggestions is hypothesized to be rooted in the human tendency to minimize the cognitive efforts for performing a (complex) task^15^, the preference of seeking confirmation over disconfirmation, the trust in the algorithm in general or in the specific outcomes^16^, and limited inputs for and capabilities of interrogating the AI and its suggestions^17^. These are because human actors have limited capacity to process information^18,19^, hence they try to ignore some informational inputs and use “mental shortcuts” to perform cognitive tasks^20^.

Under-reliance is hypothesized by the negative attitudes about AI, the strong sense of status and autonomy in making decisions by professionals, and the lack of inputs for and capabilities of interpreting the AI suggestions^21^. For instance, it is found that both low and high levels of expertise can result in algorithmic aversion, for lack of capability to appropriately understand the correct suggestions and for the strong sense of accountability for their own decisions against AI suggestions, respectively^22^.

Where over-reliance and under-reliance pose antithetical examples of inappropriate reliance^23^, the paradox of their occurrence has been one of the still unsolved dilemmas^24^. Research so far offers inconsistent insights regarding which factors can diminish these two biases. Various studies have examined the (co)relation between some factors such as the accuracy of AI suggestions^12^, the level of expertise of professionals^25^, and the possibilities for engaging in some additional reflection^13^ and the forms of reactions to AI suggestions in terms of accepting, rejecting, or ignoring^12^.

Although the overall pattern is that professionals are prone to both over-reliance and under-reliance biases^26^, current studies do not offer a consistent pattern regarding when each dynamic is more likely to happen and how they can be diminished. Current studies propose two broad categories of factors which are expected to alleviate these two biases: (1) providing explainability inputs next to AI suggestions and (2) developing a critical or balanced attitude about AI and its suggestions.

### Explainability inputs

Theories have hypothesized different impacts of “explainability” inputs in moderating the effect of algorithmic suggestions on human decisions. In particular, information processing and rational decision-making theories suggest that providing information which enables users to trace algorithmic suggestions to the specific attributes of the inputs can support them in their analytical reflection. This allows for the independent and rational analysis of the accuracy of such suggestions. Explainable AI methods can enable users to understand and interact with the explanatory inputs^27,28^. In the case of AI-based algorithms applied to medical imaging, two types of explainability inputs are relevant: *morphological* and *numerical* inputs.

Morphological inputs are mentally close to the images and anatomical regions that medical professionals are used to seeing and understanding. These inputs highlight the areas of the medical image on which the algorithm bases its findings. Such highlighting is often presented as heatmaps, also known as “saliency maps”. These heatmaps draw attention towards salient regions of the images, supporting the attentional focus of users on potentially relevant areas. However, when the algorithmic suggestions are incorrect, the heatmaps may misguide users’ attention towards areas that the algorithm has mistakenly considered relevant, increasing the risk of overlooking relevant areas^8^.

Numerical inputs include quantitative attributes of a medical case, including the general image characteristics such as density, uniformity, margin, and shape of the medical image, as well as the clinical attributes such as age and historical and genetic characteristics, which are potentially relevant for the outcome decision. The interpretation of these attributes is often based on their relative contribution to the algorithmic suggestion for each case. Presenting these attributes and their relative contribution to the AI outcome can trigger a deeper understanding of how a specific suggestion has been made by the algorithm^29,30^.

However, providing these inputs can evoke other attentional and cognitive mechanisms that can lead users to instead minimally engage in critical examination. Two mechanisms can play a role here: (1) the tendency to avoid information overload, and (2) developing confidence based on the mere presence and apparent sophistication of presented inputs. In the first mechanism, when users perform complex tasks that place pressure on cognitive resources (e.g., due to time pressure or a high workload), they may use the explainability inputs selectively and instrumentally to justify their own intended decisions (being either agreeing or disagreeing with AI suggestion). This results in a more heuristic-based usage of AI suggestions in order to compensate for the increased cognitive effort instead of systematically and critically examining the AI suggestion^31^. In the second mechanism, the very presence and complexity of the explainability inputs can create the impression of soundness of AI and its suggestion, hence causing users to follow the AI suggestions without examining their veracity^32^.

### Attitude towards AI

The second category of factors moderating the effect of AI suggestions on human decisions are attitudes towards AI^33^. Whether professionals hold a positive, negative, or ambivalent attitude towards either algorithms in general or the specific algorithm and suggestion, is considered to impact both their analytical engagement and their reliance on the AI suggestions^23^. Although a minimum level of negative attitude can trigger them to critically examine AI suggestions, carrying a strong negative attitude about AI can lead to disregarding the value of considering AI suggestions entirely and hence causing under-reliance^21^. In extreme cases, this can lead to opposition towards AI, meaning that the professionals’ attempt is focused on finding ways to disprove and counter its suggestions, regardless of their validity.

On the other hand, a positive attitude towards AI and its suggestions can trigger mental and attitudinal openness to consider such suggestions and rely on them when users deem these suggestions correct. At the same time, developing a (strong) positive attitude towards AI may cause overconfidence in its suggestions and prevent users from holding a critical, independent analytical stance, resulting in over-reliance on AI suggestions. Therefore, some scholars have hypothesized that holding an ambivalent attitude, namely both positive and negative, can overcome both biases, if professionals use the explainability inputs for a critical examination of AI suggestions.

**Figure 1:**
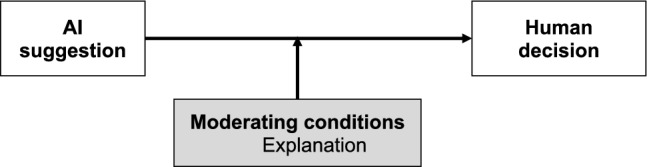
Theoretical model.

As Fig. 1 shows, we seek to answer:

*How is the effect of AI suggestion on professionals’ decisions moderated by providing different explainability inputs and attitudinal priming?*

To answer this question, we assume that when (medical) experts are provided with AI suggestions, *various dynamics* may occur, of which each can potentially cause different decisional pathways in terms of how experts engage with the task and make their decisions. This assumption is more consistent with the recent findings indicating the heterogeneity of ways in which (medical) professionals react to the same inputs, depending on how they engage with and navigate through the diagnosis process^25^.

Departing from this assumption, we argue that it is not enough that we only examine whether a specific input (e.g., incorrect AI suggestion) leads to a specific outcome (e.g., making incorrect decisions). Rather, we need to explore the various ways in which human actors interact with AI suggestions (and other inputs) and eventually make their own decision. In this view, human actors are considered as actors who have the agency to actively choose different ways of acting on the inputs and render their decisions.

Taking an exploratory approach, we examine this question in the context of conducting mammography examinations by radiologists. By analyzing 2760 decisions made by 92 radiologists, examining 15 pairs of mammography images, we show that the effect of AI suggestions on the diagnosis decisions are strong and consistent, regardless of explanation inputs and attitudinal priming. We identify distinct pathways that lead radiologists to over-diagnose, under-diagnose, and correctly diagnose. As we will show, the effects of explainability inputs and attitudinal priming are limited to some specific pathways, and cannot overcome the influence of AI suggestions.

## Methods

### Research design

The underlying research is split in two quasi-experimental studies. This approach was chosen because a lab experiment can be conducted under highly controlled conditions where accurate measurements of causal relationships are possible in an artificial environment^34^. We deployed a custom-developed online environment which allowed us to design the experiments and collect the required data. Both studies followed a between-subject design between different treatment groups. The different treatment groups are exposed either to different types of explainability inputs (Study 1) or were primed differently in relation to the capability of AI (Study 2). The participants are randomly assigned to the different groups.

#### Study 1: Participants exposed to different explainability inputs

In study 1, we experimented with different types of “explanation inputs”, namely (1) a heatmap and (2) numerical attributes of the cases. We treated both explainability methods as a gradual extension of information that explains the AI suggestion.

We designed three 3 groups:

- *Explainability-control:* no access to any explainability inputs (only supported by an AI suggestion)
- *Explainability-partial*: only able to access the heatmap only
- *Explainability-full*: access to both heatmap and case attributes.

#### Study 2: Participants primed on different attitudes about AI

In study 2, we kept all the explainability inputs, but we experimented with different modes of “attitudinal priming”. We designed 4 groups:

- *Priming-control:* received no priming
- *Priming-positive*: shown a video instruction on the effectiveness and potentials of AI tools for making medical diagnosis
- *Priming-negative*: shown a video instruction on the pitfalls and limitations of AI tools for making medical diagnosis
- *Priming-ambivalence*: show a video on both positive (effectiveness and potentials) and negative (pitfalls and limitations) aspects of AI and its outcomes.

To design the attitudinal priming in a way that represents the real-life of framing AI to participants, we edited the videos to contain positive and negative facts about AI in a medical context, presented by authorities from the field of medical AI and imaging informatics as reported in Appendix C.

### Experiment design and setup

1. **Cases**
  All participants were presented with the same sequence of 15 mammograms in the craniocaudal view on the left side of the screen and mediolateral oblique view on the right side of the screen. In both views, the left and right sides of the breast are shown (see Fig. B8, in Appendix B)^35^. These cases were selected by a panel of 3 experienced radiologists to be representative of the common cases in clinical conditions. In consultation with several radiologists, we aimed for 15 cases to provide a feasible set of tasks to be examined continuously, without requiring any break time or reducing the focus and attention due to the fatigue.
  Participants had the chance to view the images at their native resolution of 2.7 MP by clicking on them. To improve the quality of viewing, we programmed the system in a way to enforce using large enough screens, meaning the ones with a minimum resolution of 960 × 540 pixels. Participants also had the chance to “enlarge” the images and “Zoom” on each part of the image. The cases were shown to the participants one after the other and they were not able to go back or change the order of the cases. See Appendix D2 for the mammograms, and see Appendix D1 for details on classification, lesion types, and lesion sizes The participating radiologists had to classify the mammograms based on the BIRADS classification system, used as standard in the assessment and reporting of mammograms^36,37^.
2. **AI suggestions**
  To examine the influence of the given AI suggestions on the way participants interacted with the cases and made decisions, we intentionally provided AI suggestions in a way that, in 7 cases, the AI makes a correct BI-RADS classification on both breast-sides. In 8 cases, we intentionally created incorrect AI suggestions on only one breast-side (balanced on left and right breast-sides), with minor (1 point error) and major (2 points error) deviations from the ground truth, equally balanced in terms of “over'' and “under” diagnosis. This way, out of 30 suggestions (2 suggestions per each mammogram), we provided 8 incorrect suggestions (27%) to represent enough incorrect suggestions but not too many to create a mistrust in the suggestions entirely. Because of the clinical interchangeability of BI-RADS score 1 and BI-RADS score 2, the choice was made to solely include cases with BI-RADS score 2 to prevent ambiguity between participant’s answers. The following Figure shows the order of the cases and the AI suggestions. We also designed an equal number of “over-diagnosis” vs. “under-diagnosis” AI suggestions (4 each) to be able to control for the effect of this factor on the outcome decisions of radiologists. We intentionally aimed for more small errors (6) than large errors (2) in terms of AI suggestions to represent realistic scenarios where the chance of offering large errors is smaller than the chance of committing small errors. Having many big errors could create major mistrust in the AI suggestions and potentially ignoring them.
  AI suggestions were not visible to the participants, unless they clicked on the “Show AI suggestion” button (see Fig. A1, in Appendix A). The decision to display the AI predictions at the click of a button was made to measure how fast participants decide to incorporate the help of the AI in their classification. In order to collect this data, a timer was started at the beginning of each mammogram, which measured how long the respective participant needed to press the "Show AI suggestion" button for the respective mammogram. The frequency of each BI-RADS category in ground truth and AI suggestions are balanced (see Fig. 2).

**Figure 2:**
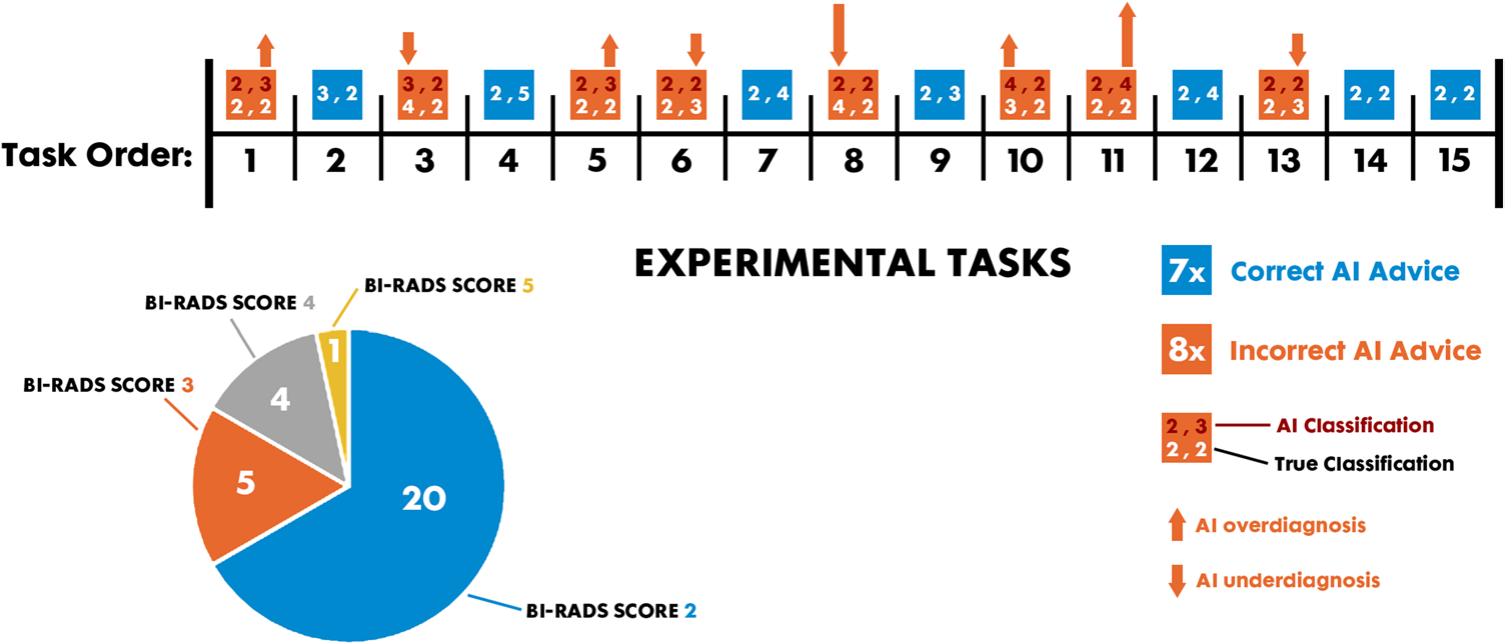
Mammogram cases and the associated true and AI classifications.
3. **Classification input**
  Participants were presented with the BI-RADS classifications scale (only 1–5) for each breast and were asked to provide their BI-RADS decision on a breast-side level. BI-RADS category 0 is left out because the experiment only allows complete assessment; BI-RADS category 6 is left out because this category requires a biopsy of suspicious tissue, however, this is not related to this research project which just covers image recognition with the help of AI. We traced how fast they gave their first classification and how frequently they changed their decision before submitting each case.
  Truth was determined from the original clinical reports (which are already double-read) and a third confirming reading by an independent experienced reader. Since our study focuses only on BI-RADS assessment (not "real diagnostic performance in detecting malignancy"), we did not distinguish between benign vs. malignant. The measurable lesions had an average size of 15.75 mm (SD = 7.68, see Appendix D1). We did not control for the number of lesions, since the focus was on assigning a BI-RADS score to each breast-side.
4. **Heatmap**
  The "artificial" heatmaps were drawn by a senior radiologist based on identifying the most likely area of the mammogram to be considered as pathological and thus provide participants with an opportunity to better understand the process behind the respective AI outputs. Heatmaps were hidden as default and participants could see them by clicking on the “Show Heatmap” button. The green area of the heatmap indicates that the AI expects no malignant tissue in the respective region, while the yellow area indicates a low probability for malignant tissue. The red area indicates a high probability for malignant tissue in the respective regions. The button "Hide Heatmap" could be used to close an opened heatmap. Again, to record to what extent the participants engaged with the additional information conveyed by the heatmap, we measured how often they switched the heatmap on and off for each case. In addition, the time until the heatmap was first opened per case as well as the total time the heatmap was actually opened per case was measured. See Appendix D3 for the heatmaps.
5. **Case attributes**
  We used a set of clinically relevant case attributes to provide the participants with numerical explanations on which parameters of the image contributed to the AI suggestion in that particular case (see Appendix D4). These attributes explain individual predictors with respect to a set of high-level concepts based on their importance to a particular model outcome^30^. Case attributes are often presented in terms of “relevance pooling bar charts” for specific cases or the entire sample^29^, or in terms of domain-specific natural language^30^. Due to the fact that abnormalities in mammograms are assessed on the basis of certain superordinate categories^38^, the imitation of case attributes is relevant for providing the Explainability-full group as well as all priming groups with an additional explainability method next to the heatmap. If a mass is seen in a mammogram, it is evaluated based on three descriptions: shape, margin, and density^38^. In addition, detector uniformity is an important parameter in digital mammography to guarantee a level of image quality^39^. Together with the age and genetic predisposition of the patient, the descriptor's shape, margin, density and the parameter of uniformity are all used as high-level concepts that act as a certain imitation of case attributes. As a form of representation, a bar chart was chosen, whereby the associated bars indicate to what extent the single case attributes hypothetically influenced the AI suggestion. All case attributes were carefully characterized by a senior radiologist who is also experienced in developing and using deep-learning algorithms. The senior radiologist rated the case attributes per case from -3 (indication for benign finding) to 3 (indication for malignant finding), whereby the bars in the bar charts represent the ratio between these ratings. Values from -3 till -1 are represented as bars to the left (indication for benign finding) and values from 1 to 3 are represented as bars to the right (indication for malignant finding). The case attributes could be accessed by hovering the mouse over the information field above the "Submit answer & continue" button (See Appendix A1, No. 5). We measured how often and for how long a participant opened the case attributes for each case. The case attributes were automatically closed again after they had been open for 10 s continuously. This design decision was made to counteract the potential behavior of participants who keep the case attributes continuously open without paying attention to them.
6. **Case submission**
  The “Submit answer & continue” button registers the given BI-RADS classifications in and forwards the participant to the next mammogram. The participants haven’t had the opportunity to go back.

#### Experiment process

**Figure 3:**
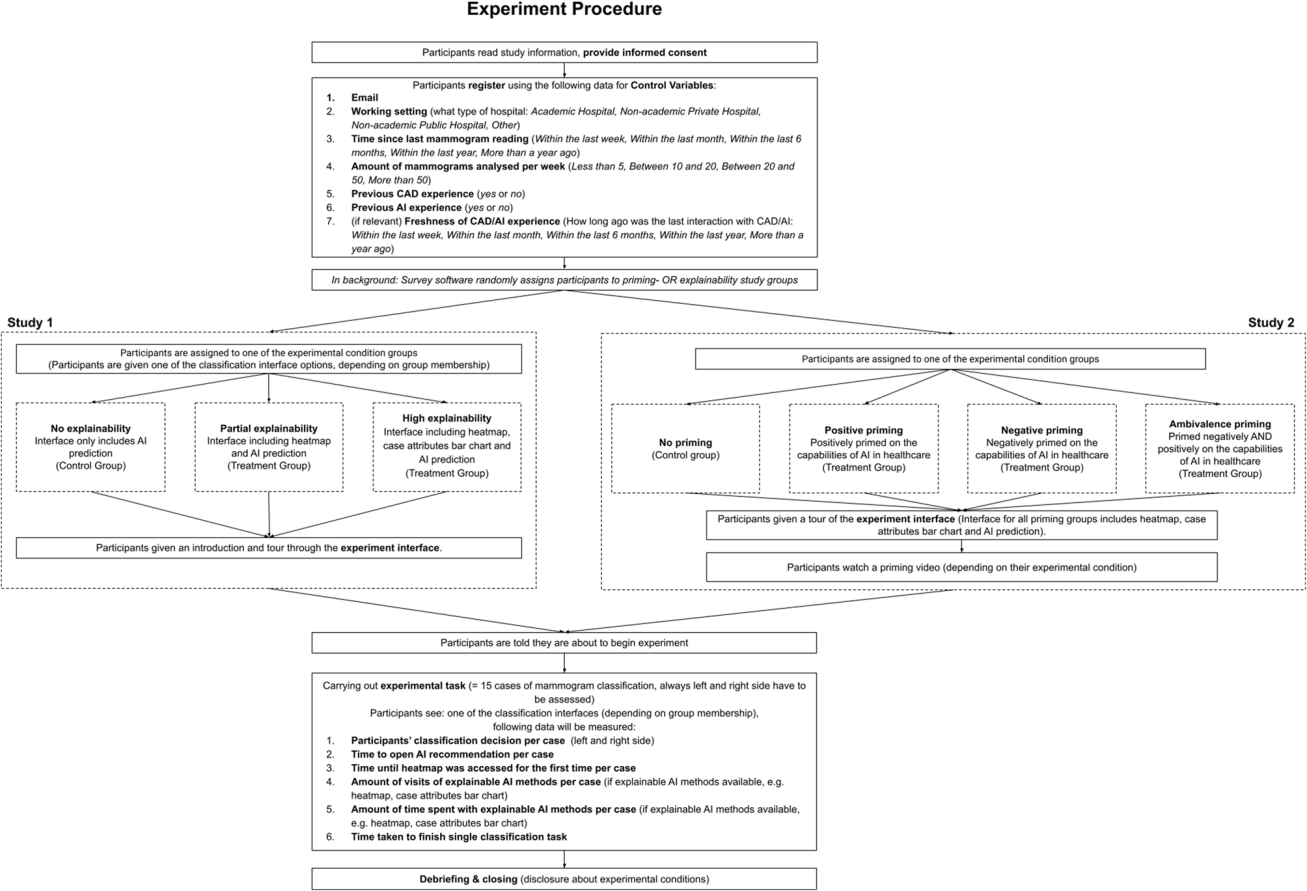
Experiment procedure.

As Fig. 3 shows, all participants were recruited via the same introduction page where they were informed about the study purpose and were asked for their consent for their data being used anonymously for the study (see Appendix B for more details). While the participants were introduced to the experimental task, they were explicitly told that they would be supported by a real AI during the experiment in order to avoid suspicion, which could distort the participants' answers. Furthermore, it was clearly stated before the start of the experiment that the data of the participants will be treated with the utmost confidentiality and that it will only be used for research purposes. Next, the participants were randomly distributed to one of the sub-groups of each study to receive their own specific interventions (either to Study 1 or Study 2). All participants received an introduction through the experiment interface to make them familiar with the classification tasks. Subsequently, If they were assigned to one of the priming groups (Study 2), they had to watch a related priming video (positive, negative, and ambivalence).

### Sample and participants

We recruited a total of 92 radiologists who were trained and experienced in performing BI-RADS classification. Through multiple channels, such as professional communities and personal contacts, we distributed the invitation for the study among radiologists worldwide. Eventually, 34% of the participants were from Europe and 66% were from Iran. To incentivize them, we offered participants the chance to win a free ticket to one of the upcoming conferences in radiology (annual meeting of the European Society of Medical Imaging and Informatics, EuSoMII). We asked the participants about their experience of performing mammography (overall experience and recent experience) as well as their experience in terms of using AI/CAD tools (overall experience and recent experience). As Fig. A2.b shows (Appendix A), more than half of the participants had the experience of conducting a mammogram examination “within the last week” at the time of conducting the experiment. For only 12% of the participants, the last mammogram reading was more than 1 year ago. This indicates a certain “freshness” of reading mammograms within the participant pool. However, the frequency of reading mammograms is rather low across the participating radiologists, whereby more than half read less than 10 mammograms on a weekly basis and only 26% more than 20 mammograms (see Fig. A2.a, in Appendix A). Furthermore, 71% of the participants stated that they have no previous experience with CAD/AI (see Fig. A2.c, Appendix A). This indicates that the participants generally had limited experience in terms of using CAD or AI tools at their work. Additionally, less than half of the participants who had some experience with CAD/AI tools, had such experience within the last 6 months before the experiment (see Fig. A2.d, Appendix A).

### Ethical considerations

The study obtained the approval of the ethical committee of the department of the principal investigators by carefully examining the potential ethical and legal consequences. The research was conducted outside of the clinical context and did not impact any clinical decisions on specific patients. Informed consent was obtained from all participants in the research. All participants participated in the research on a volunteer basis and they were informed about the purpose of the study and what they are going to do in advance. In addition, they all had the chance to actively “opt-in” for participating with the condition that their data would be used for the research. All the obtained data were immediately anonymized at the start of the analysis. All methods of collecting and analyzing data and conducting research were performed in accordance with the relevant guidelines and regulations.

### Data analysis

#### Operationalization of concepts and analysis process

The operationalization of concepts and analyses were at the different levels of analysis, namely (1) mammogram level (both entire mammogram and each breast-side), (2) participant level, and (3) study group level. For each participants, we obtained 2 decisions on each mammogram (one BI-RADS scoring per each breast-side), resulting in (2*15) 30 decisions at the breast-side level (total 92 participants * 30 = 2760 data points). When we aggregated some factors as the level of mammograms (e.g., the average of the decision errors on the left and right breast-sides or the total time spent on the mammogram), we reported this data and analysis at the mammogram level (hence having 92*15 = 1380 data points). Given the exploratory character of the study, we adhered to the principle of capturing and describing heterogeneity in the phenomenon and did not oversimplify the patterns based on single-model analysis^40^. This way, we avoided potential fishing of (statistically) significant patterns which may not be meaningful. Hence, we constantly iterated between the empirical patterns and the specific cases and used the granular data on the patterns of actions performed on the cases to (1) identify the heterogeneity of the patterns and (2) examine various (rival) explanations^40^.

#### Outcome variables

We operationalized our outcome variables based on the final decision performance of the human actor compared to the ground truth of the underlying BI-RADS category for the mammogram as well as the given AI suggestion (deviation of BI-RADS class given by the human actor from the BI-RADS class that is considered as the ground truth and given by the AI, see Table A2).

#### Control variables

Two categories of control variables were (1) the level and recency of experience in performing BI-RADS classification and (2) the level and recency of experience with using CAD/AI tools. The first control variable that was deemed as closely related to the engagement with the tasks and interacting with AI suggestions was (1) recent experience of classifying mammograms. It might be that radiologists who just recently read an increased number of mammograms will be able to identify abnormalities in a mammogram more accurately and quickly in a substandard artificial clinical setting without additional cognitive exertion owing to their routine.

The second control variable that was deemed as relevant was the experience of using CAD/AI tools. This variable was also added in consultation with one of the involved senior radiologists, who noted that a lot of radiologists had bad experiences with CAD systems in the past and could therefore be negatively preoccupied with AI. This could have caused greater distrust towards the underlying pseudo-AI among radiologists who already had experience with CAD and they may have had a negative attitude towards AI upfront.

#### Analytical pathways for making the decision

In addition, we explored the “analytical pathway” through which participants navigated through the tasks and interacted with the various informational elements. This concept emerged as an important way of explaining “how” participants engaged with the tasks. In particular, we analyzed the sequence of viewing “Heatmap”, consulting “AI suggestion”, and making “Decision” in performing each task. We also paid attention to the timing of such sequences in order to understand different pathways and analytical journeys of participants. By crossing these analytical pathways with different groups and outcomes, we tried to make nuance interpretations of the theoretical relations.

## Findings

### Participants’ engagement with the tasks

#### Time spent on the tasks

On average, participants spent 1.3 min per mammogram, with a standard deviation of 71 s, which is comparable with the clinical reading time^26,41^. There is no significant difference between the various groups in terms of the time of performing tasks. Nevertheless, the accuracy of decisions is significantly higher for cases in which radiologists spent more than the average time, compared with those conducted less than the average time (p-value = 7.714e−05). During the experiment, the average time spent on tasks slightly reduces (no statistical difference).

#### Consulting AI suggestions

Participants consulted AI suggestions in 84% of the tasks, with a tendency to do so in the second half of the time of conducting the examinations. Accessing AI suggestion was not correlated with lesion type (Χ^2^ (3,* N* = 1380) = 1.8, *p* > 0.605).

#### Consulting heatmap

In 83% of the cases where heatmap was available, the participants consulted the heatmap and on average kept the heatmap open for 13% of the examination time. Heatmaps were rather evenly consulted at any time during the tasks.

#### Consulting case attributions

When it was available, in 9% of the cases participants consulted the “case attributions” longer than 2 s (to be considered as meaningful viewing), which showed that this informational element was not consulted at the level that can be influencing their decisions. There is a strong association between the number of times that the case attributes are visited and the accuracy of the diagnosis.

#### Sequence of consulting AI and heatmap

We examined the sequence of consulting AI suggestions and heatmap, before making the final diagnosis decision. The most frequent pattern was related to (52%) first consulting the heatmap and then AI suggestion, followed by only consulting AI suggestions (23%), first consulting AI and then examining heatmap (9%), only consulting heatmap (8%), and skipping both AI suggestions and heatmaps (8%).

### The effect of AI suggestion on human decision

#### Overall accuracy of human decisions and diagnosis types

**Figure 4:**
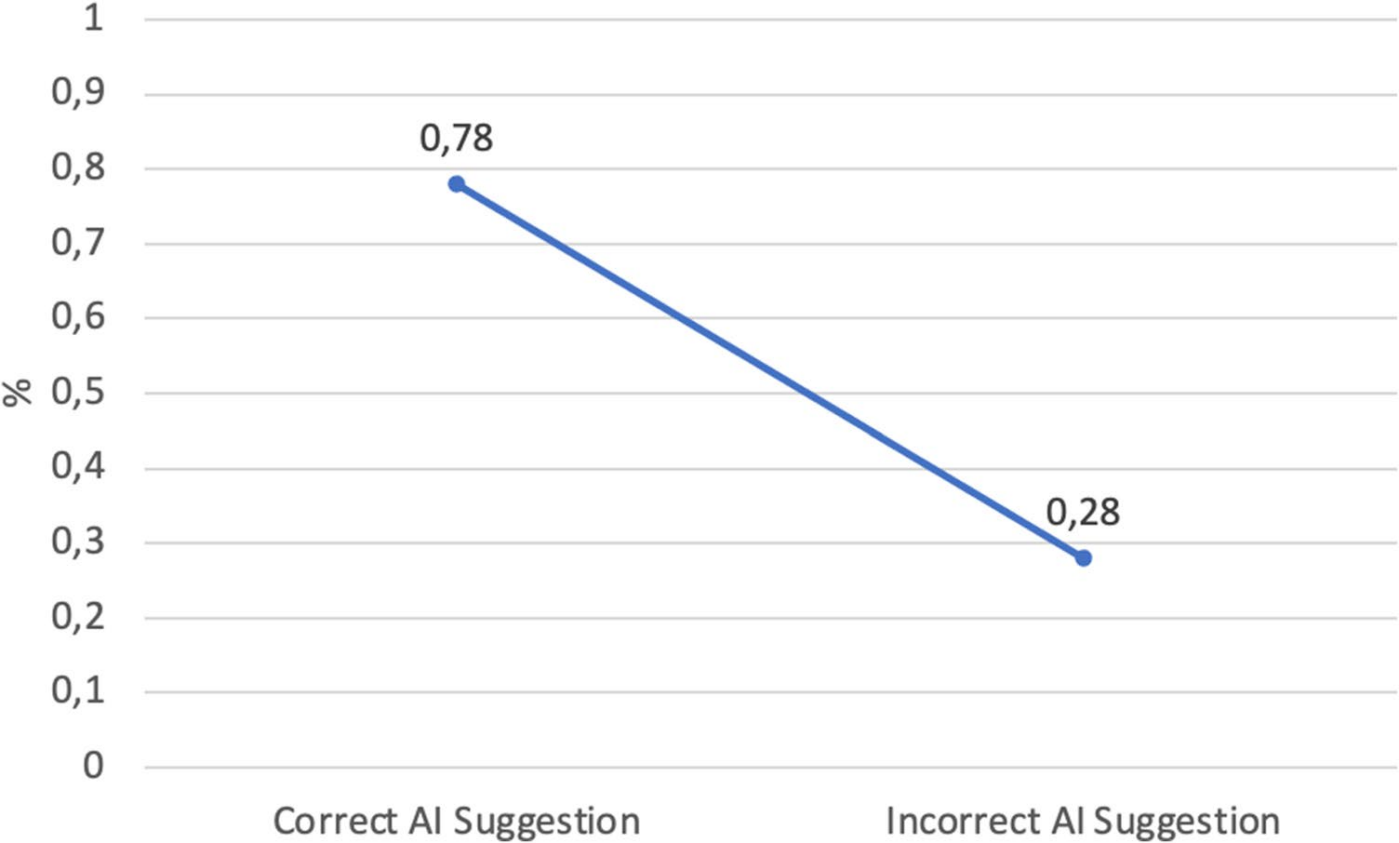
Difference between radiologists’ correct diagnosis when receiving correct and incorrect AI suggestions (breast-side level).

At the most granular level, each task involved making two decisions: scoring left and right beast sides. On 2760 decisions, radiologists made 64% correct decisions, which is lower than the overall accuracy of AI suggestions (73%). Radiologists have a tendency towards over-diagnosis (20%) than under-diagnosis (16%). When AI suggestions are not consulted, the share of correct decisions is 61% and when AI is consulted, it is 65%. As Fig. 4 shows, the average accuracy of human decisions drops from 78% when AI offers correct suggestions to 28% when AI offers incorrect suggestions (p-value: 2,2 e−16).

**Figure 5:**
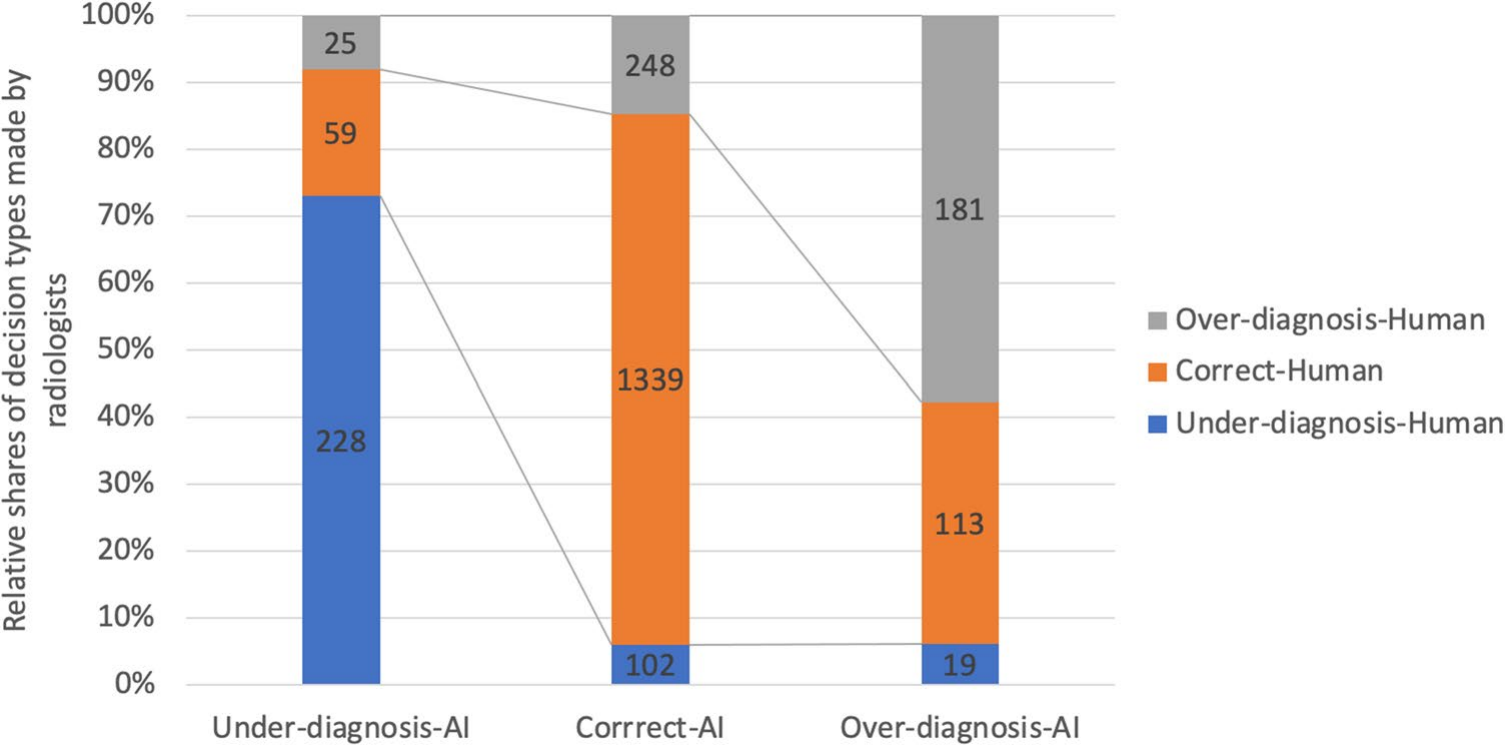
Coincidence between the types of radiologists’ decisions and AI suggestions.

The type of decision made by radiologists has an agreement of 73%, 79%, and 58% with the type of AI suggestions for “under-diagnosis”, “correct”, and “over-diagnosis” (see Fig. 5). The share of “appropriate disagreements” to the total disagreements with AI suggestions is 30%. When radiologists disagreed with AI suggestions, the chance of making incorrect decisions was 70% (compared with the average of 36% incorrect decisions). The share of “inappropriate agreement” to the total number of agreements with AI suggestions is 23%.

#### The pattern of human decisions across the mammograms

**Figure 6:**
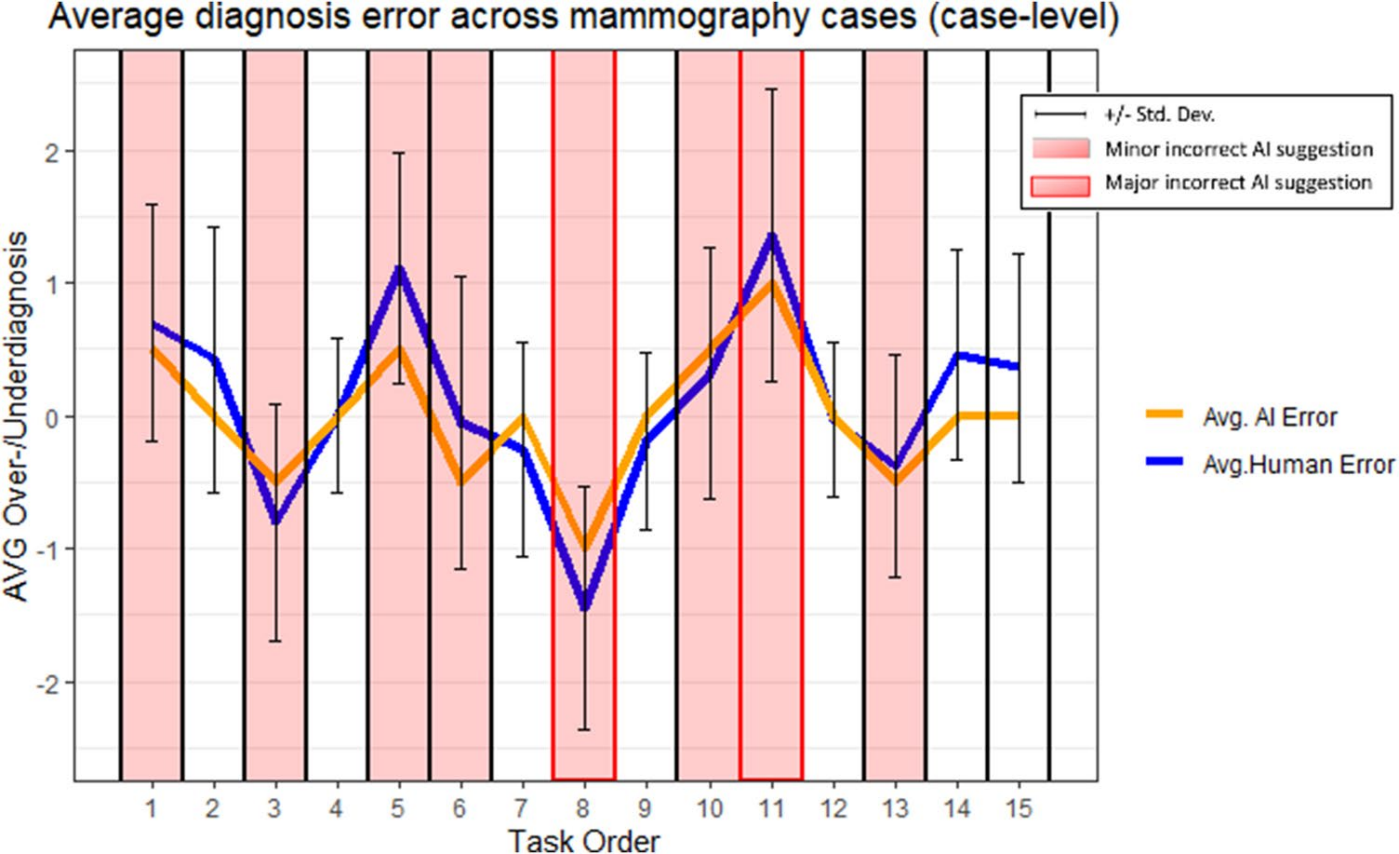
Pattern of average diagnosis error across mammography cases (case-level).

To further examine the pattern of decisions, we mapped the average human error (considering over- and under-diagnosis) across the 15 mammograms. As Fig. 6 shows, the average of human error matches the type and size of errors of AI suggestions.

**Figure 7:**
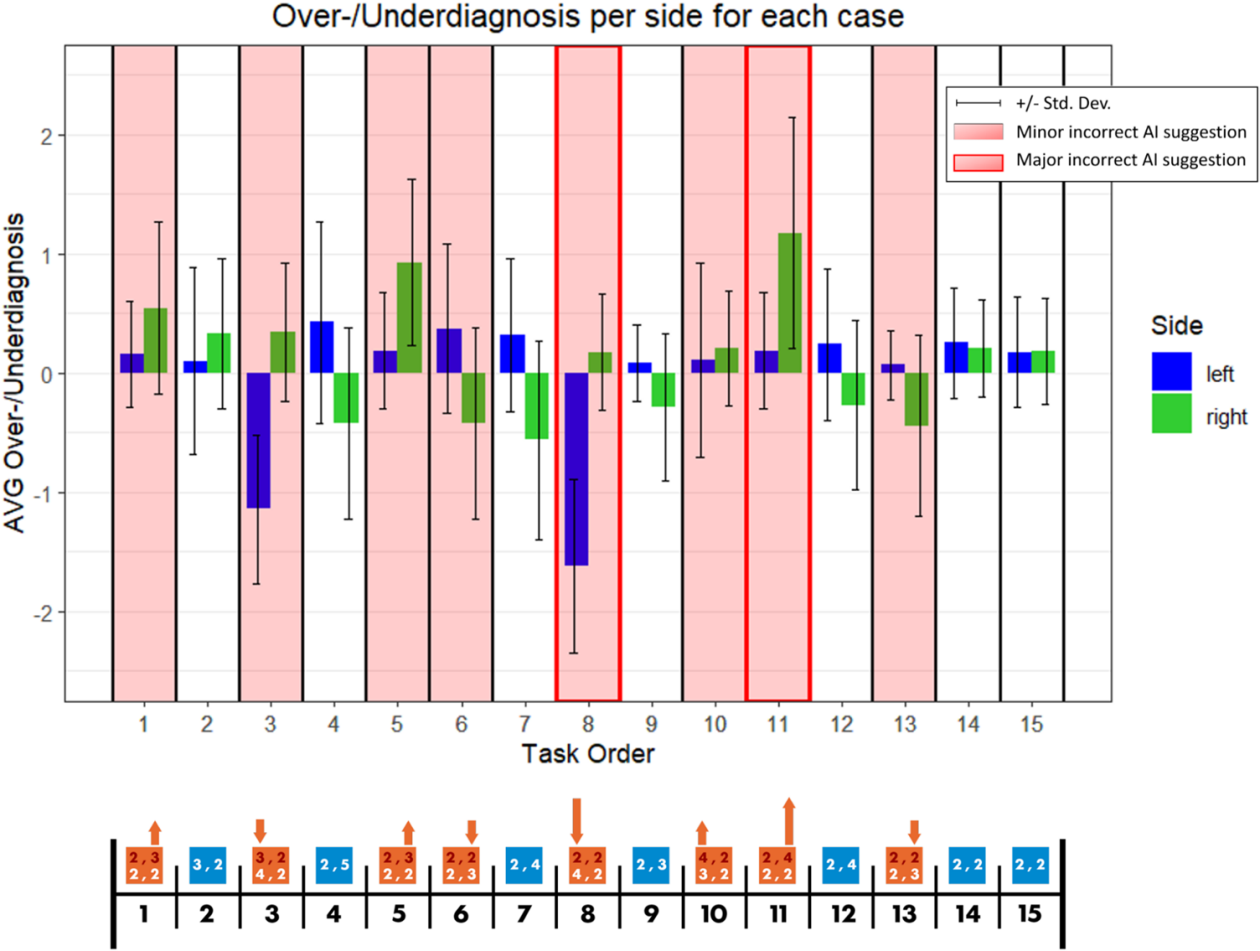
Pattern of diagnosis errors based on mammograms (breast-side level).

This pattern is more vivid when we look at each breast-side (see Fig. 7). The errors of human decisions match the AI suggestion errors, in 6 out of 7 cases where AI offers wrong suggestions. The highest human errors correspond to the situations where AI suggests major over-diagnosis (Case8-left-breast) and major under-diagnosis (Case11-right-breast). Case 10 (left breast) seems to be an exception in the sense that the over-diagnosis suggestion on the left breast is not reflected in the human decisions.

### The effect of explainability inputs

There is no significant difference across “explainability-control”, “explainability-partial”, and “explainability-full” groups in terms of the diagnosis errors of radiologists.

### The moderating effect of attitudinal priming

There is also no significant difference across the four groups in study 2, where they received different types of attitudinal priming. In addition, there is no significant difference between the average of human error across the different levels of experience with AI/CAD (Table 1) and the experience of conducting mammography examination.

**Table 1:** Difference in diagnosis errors between participants with low or high e*xperience with AI/CAD.*

	Low experience with AI/CAD	High experience with AI/CAD	p-value
Mean of human error (SD)	0.05 (0,83)	0.06 (0,84)	0.8171

### Pathways of “correct”, “over-diagnosis” and “under-diagnosis” decisions

**Figure 8:**
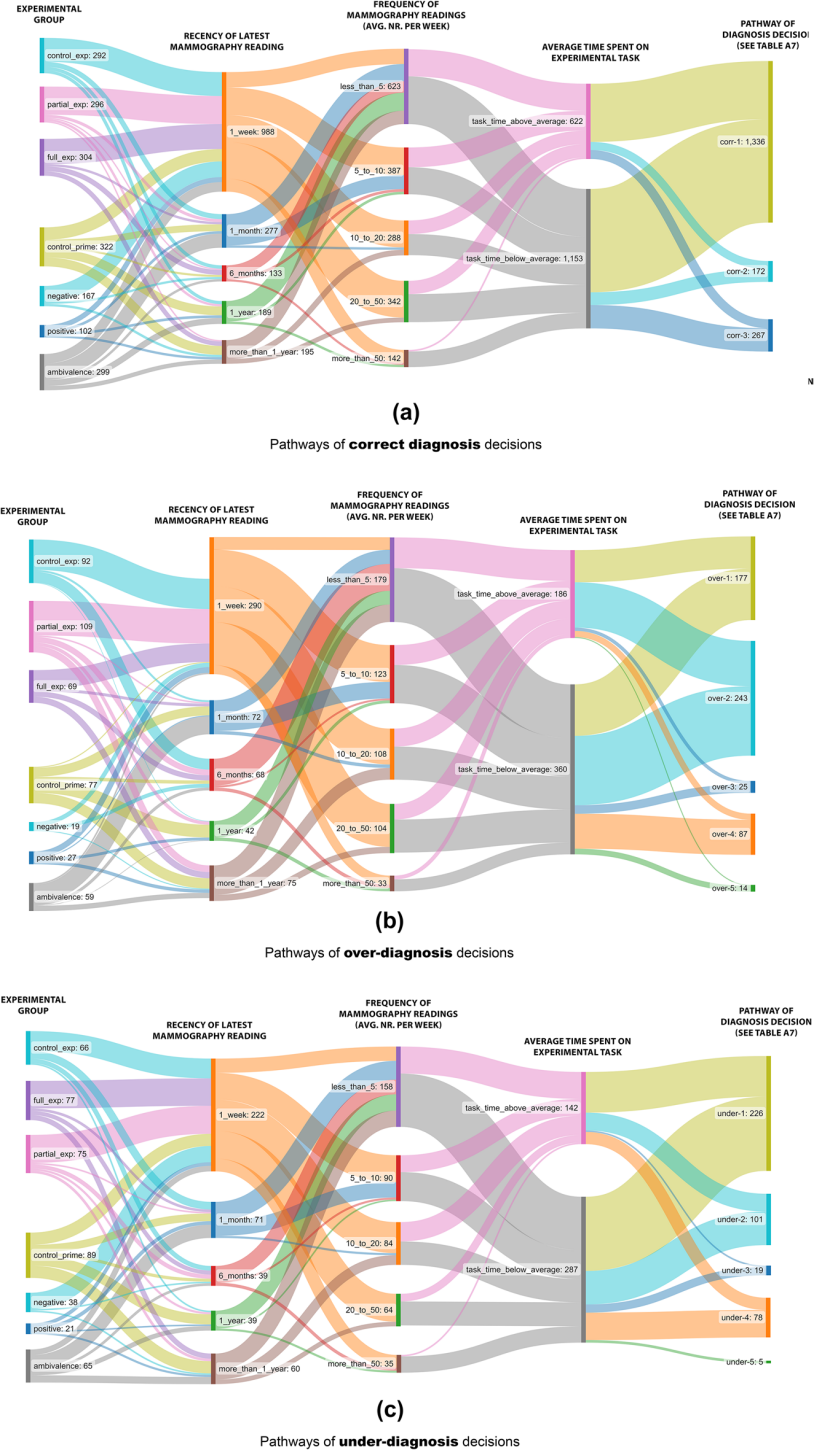
Pathways of (**a**) correct, (**b**) over-diagnosis, and (**c**) under-diagnosis decisions. In these Sankey diagrams, the first column depicts which experimental group participants were in, where the 3 top groups represent the explainability groups (study 1), and the bottom 4 groups represent the attitudinal priming groups (study 2). The second column represents how recent participants have the experience of conducting their latest mammography reading. The third column represents how many mammography readings a participant had performed per week on average. The fourth column represents whether a participant spent overall more or less than average time on the experimental tasks. The fifth column shows which pathway of a diagnosis decision is followed.

Noticing the strong association between AI suggestions and radiologists’ decisions, it is helpful to explore the pathways that are associated with correct, over- and under-diagnosis decisions. For each of these decision types, there are various pathways depending on the type of AI suggestion and whether radiologists consulted AI suggestions (see Fig. 8 and Appendix G for more details).

#### Pathways of making correct diagnosis

The correct decisions made by radiologists (64%) can be mapped to 3 pathways.

The most dominant pathway (Corr-1) is when the AI suggestions are correct *and* radiologists consult AI suggestions (76%). This pathway happens across all groups, though is most popular in the “Priming-positive” group. These cases are relatively more popular when the true scores are “2” (34%) or “5” (37%) than being in the middle of the range, “3” (11%) or “4” (18%). There is no pattern regarding the average time spent on the task, the level and recency of experience, or experience with using CAD/AI tools.

The second pathway (Corr-2) is when AI offers an incorrect suggestion and radiologists consult such suggestion, yet they make a correct decision (13%). This pathway is observable in all groups and has the highest relative popularity among least (less than 5 examinations per week) and most (more than 50 examinations per week) experienced radiologists. This pathway never happens when the true score is “5”. Overall, the average time spent on these tasks (89 s) is higher than the total average (79 s).

The third pathway of making correct diagnosis (Corr-3) is when radiologists do *not* consult AI suggestions (15%). These cases were present in all groups, with a lower relative frequency in Priming-positive (1%) and Priming-negative (6%) groups, and higher relative frequency in Explainability-control (27%) and Priming-ambivalence (25%) groups. These cases are more popular when radiologists have a moderate level of experience (5 to 20 mammograms per week, 48%) or had a recent experience of conducting such examinations (in the last month, 53%). This pathway is mainly visible in clearly normal (true score “2”, 44%) or clearly abnormal (true score “5”, 27%) cases. The average time spent on these tasks (63 s) is lower than the overall average (79 s).

#### Pathways of making over-diagnosis

From all the cases that radiologists make an over-diagnosis of 1 or 2 scores, in one third of them they consult AI suggestions, which also indicates an over-diagnosis (Over-1). This happens in all groups and there is no pattern in terms of the experience with AI/CAD nor the frequency or recency of performing such examinations. However, this pathway is only observed in cases with a true score of “2” (82%) or “3” (18%) and overall, the average time spent (113 s) is higher than the overall average (79 s).

The most common pathway (Over-2) for making an over-diagnosis decision is when AI offers a correct suggestion but radiologists consult AI suggestion and deviate from it towards a higher score (46%). In 87% of these cases, the true score is “2”. There is no other pattern regarding the experiences with AI/CAD or conducting examinations. The average time spent on these tasks (95 s) is higher than the overall average (79 s).

In 5% of cases, AI suggestions is an under-diagnosis, yet radiologists go in the opposite direction and make an over-diagnosis (Over-3). These cases are present only in mammograms “6” and “13″ where AI deems both breast-sides as normal, although the cases had some issues. These cases were present in all groups, except Priming-control and Priming-positive”, and did not happen when radiologists had a high level of experience (more than 50 per week). In all these cases, the participants spent less than average time on performing the tasks (59 s on average).

In 17% of the cases, radiologists did not consult AI suggestions and made an over-diagnosis of 1 or 2 scores (Over-4). These cases were mostly popular in Priming-ambivalence and Explainability-partial groups, and never happened when radiologists were negatively primed. These cases never happened when radiologists had a high level of experience (more than 50 per week). Almost all these cases were related to situations where the true score was “2” (80%) or “3” (19.5%). On average, these cases were conducted in a shorter time than the average time (59 s).

Finally, in 7% of the cases, radiologists made an over-diagnosis of 3 points above the true score (Over-5). These cases only happen when radiologists had no experience of conducting mammography in the last 6 months, and on average, the time spent on these tasks is above the average (95 s).

#### Pathways of making under-diagnosis decisions

In very few cases (5 out of 2760) radiologists made an under-diagnosis of 3 points below the true scores (Under-5). For the rest, the most popular under-diagnosis pathway (Under-1, 54%) is when AI offers an under-diagnosis and radiologists consult such a suggestion. These cases are rather evenly popular in all groups, across different levels of experience (both frequency and recency). On average, these cases were examined in a comparable time as the overall average (75 s).

In one-fourth of the under-diagnosis decisions, radiologists consulted the correct AI suggestions, yet they deviated towards lower scores (Under-2). These decisions were made when radiologists were experienced (at least 5 examinations per week, 95%) and they were not positively primed (96%). This deviation is more likely to happen in the cases with a high level of true score (“5” or “4”). On average, these cases were examined in a comparable time as the overall average (82 s).

A small number of under-diagnosis cases (4%) relate to conditions that AI is offering an over-diagnosis suggestion, yet radiologists take the opposite direction and make an under-diagnosis decision (Under-3). This situation happened only in the mammograms #10 and never happened when radiologists had an intensive activity of doing mammography (more than 50 per week). On average, these cases were conducted in a longer time (98 s) than the average.

Finally, in 18% of the under-diagnosis cases, radiologists did not consult AI suggestions. There is no specific pattern in terms of the groups and task-related factors (Under-4). In 74% of these cases, the participants did not have any experience with AI/CAD before. On average, these tasks were conducted in 67 s.

## Discussion

Overall, our findings show that radiologists’ decisions follow AI suggestions in terms of the error size (number of points on BI-RADS scale) and type (correct, over-, and under-diagnosis). The chance of making correct diagnoses is 78% when consulting correct suggestions and the chance of making incorrect diagnoses is 72% when consulting incorrect decisions. This pattern is visible across all the 15 tasks and especially the specific breast-sides on which the (incorrect) suggestions are offered. This confirms prior research showing that the presence of algorithmic suggestions can reduce the accuracy of diagnostic decisions^4–7,26^ especially when such suggestions are incorrect and limit the focus and analytical engagement of human actors.

Overall, the diagnostic error was not significantly different across experimental groups, participants with different levels of experience in performing the task (both frequency and recency) and experience with CAD/AI tools.

Regarding the explainability inputs (Study 1), we see that participants sparingly used the numerical inputs (case attributions), yet they consulted the heatmap frequently and did so often before they consulted the AI suggestion. Our findings show that explainability inputs are not entirely overlooked^42^. In fact, when these inputs are close to the mental models of experts, they are consulted and used as heuristics for reflecting on the case and the algorithmic suggestion. This confirms the explanation that professional experts tend to use the forms of explanation that are closer to their mindsets and require less cognitive effort for understanding them^31^. This can also be because heatmaps offer a form of cognitive short-cut for radiologists to focus on specific morphological areas of the image and to ignore the other parts, which might be a reason for the tendency towards over-diagnosis than under-diagnosis^8^. We also did not see any specific pattern showing that tasks performed with consulting AI suggestions took a shorter time than those performed without consulting AI suggestions.

As for attitudinal priming (Study 2), we do not see a clear effect on the diagnostic decisions. The effect of these interventions appear in some specific pathways. This can be partly because participants had an overall limited experience with CAD/AI tools, but were generally experienced in performing the tasks. Therefore, triggering them to be positive and/or negative towards such algorithmic tools in general was not comparable with the effect of the factual inputs that they interacted with (the images, heatmaps, and suggestions).

The various pathways leading to correct, over- and under-diagnosis show that there is no single pattern that can be generalized to all situations. To arrive at correct diagnoses, radiologists may confirm correct AI suggestions (Corr-1, 76%), they also may manage to appropriately disagree with incorrect suggestions (Corr-2, 13%) especially when they spend extensive time. They can also make correct decisions without consulting AI suggestions (Corr-3, 15%) when the cases are obviously normal or obviously pathological. Even with correct AI suggestions, radiologists may spend above average time on the cases to find some issues (over-diagnose), when they are not primed to be positive about these suggestions.

Finally, to arrive at under-diagnosis decisions, the most common path is consulting an under-diagnosis AI suggestion (Under-1, 53%). However, radiologists who have frequent experience and who are not positively primed may spend extensive time to disprove correct suggestions, particularly when these suggestions concern high BI-RADS scores (Under-2, 24%). Finally, spending limited time on a task while ignoring AI suggestions and spending a relatively short amount of time examining the heatmap, together lead to missing pathologies in the cases and lead to under-diagnosing decisions (Under-4, 18%).

## Limitations and future research

Several conditions that limit the generalizability of our findings and hence call for future studies.

### Participants

Generally experienced participants, but limitedly experienced in terms of using CAD/AI tools are representative of the majority of the current radiologists^43^. These participants have limited experience in terms of how to react to AI suggestions and interrogate them. Hence, it is important to investigate how the experience of working with these algorithms impacts the way radiologists make decisions.

### Cases

Having 15 cases, with rather uneven distribution of error types, did not permit for systematic comparisons between different case types (e.g., difference in mass, or calcification) or different types of AI suggestions (e.g., small vs. large errors). Also, uneven distribution of correct and incorrect cases limited a systematic analysis of their effects within (sub)groups. “BI-RADS classification” represents a structured decision with objective decision categories^36^. Although many current AI applications focus on these forms of tasks^44^, there are other forms of algorithms which offer inputs for making less structured decisions, such as segmenting certain lesions and offering measurements that are not the final decision, but rather are part of the informational inputs for making a medical decision. This different role of AI in the medical decision-making process can potentially trigger distinct ways of interacting with them and using them.

### Setting

Our experiment was conducted outside of a clinical setting because the experiment application was made available online and allowed participants to participate at any time and from any location with their own private equipment. The monitors that are typically used in a clinical setting to analyze mammograms are technically superior to traditional monitors. This may have underrepresented fine abnormalities such as microcalcifications. We attempted to mitigate this limitation by adding a zooming option, but the "low-resolution" restriction remained. In addition, the online experiment limited the possibility of controlling for the environmental distractions and ensuring a comparable background light as is provided in the clinical reading rooms.

## Supplementary Information

Supplementary Information 1.

Supplementary Information 2.

Supplementary Information 3.

Supplementary Information 4.

Supplementary Information 5.

Supplementary Information 6.

Supplementary Information 7.

## Data Availability

The datasets generated during and/or analyzed during the current study are available from the corresponding author on reasonable request.
