# Supplementary material for: The impact of AI suggestions on radiologists’ decisions: a pilot study of explainability and attitudinal priming interventions in mammography examination
Source: Sci Rep. 2023 Jun 7;13:9230. doi: 10.1038/s41598-023-36435-3 (PMC10247804; doi:10.1038/s41598-023-36435-3)
Supplement: Supplementary file 1 — Supplementary Information 1. [file 41598_2023_36435_MOESM1_ESM.docx]

Appendix A - Figures & Tables

**Table A1.** Participant groups and their associated interventions

|  |  | **Explanation inputs** | | **Attitudinal priming** | |
| --- | --- | --- | --- | --- | --- |
|  | **participant_type** | **Heatmap** | **Case attributions** | **Positive priming** | **Negative Priming** |
| Study 1: Different explanation inputs | Explanation-Control |  |  |  |  |
|  | Explanation-Partial | ✓ |  |  |  |
|  | Explanation-Full | ✓ | ✓ |  |  |
| Study 2: Attitudinal Priming | Priming-Control | ✓ | ✓ |  |  |
|  | Priming-Positive | ✓ | ✓ | ✓ |  |
|  | Priming-Negative | ✓ | ✓ |  | ✓ |
|  | Priming-Ambivalence | ✓ | ✓ | ✓ | ✓ |

**Figure A1.** Experiment Interface (for classification tasks, numbers within the pictures are elaborated in the main text)
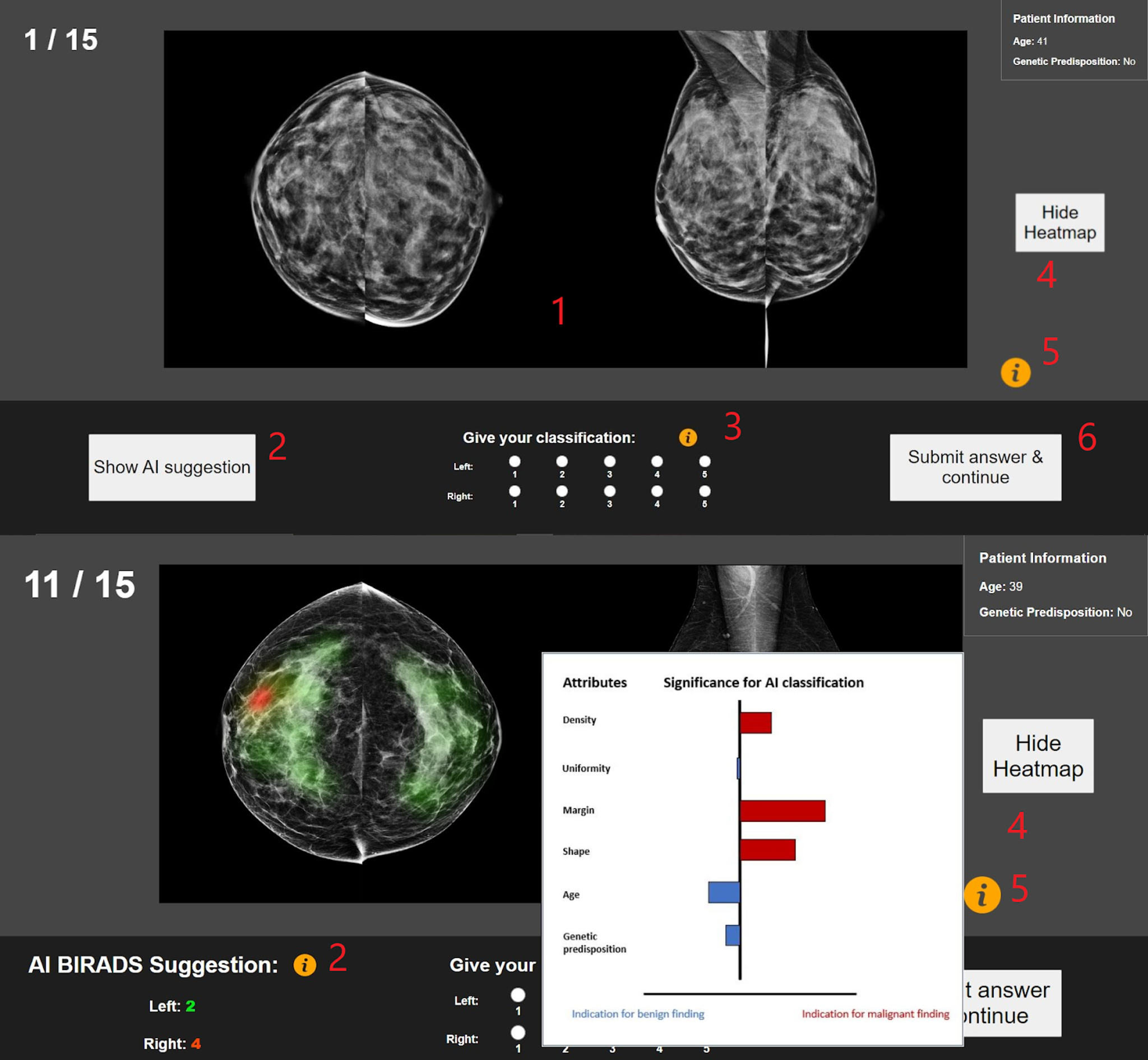


**Figure A2.** (a) Actual implemented heatmap method (Grad-CAM method) for mammograms showing breast cancer (heatmaps were generated by a senior radiologist using Affinity Photo V1.4, <https://affinity.serif.com/photo/>) and (b) actual case attributes showing the feature-wise contributions of the input variables ‘Composition’ and ‘Age’ for an undefined prediction. Contrastingly, in the bottom line, (c) a heatmap from the underlying experiment is shown as well as a (d) case attribution bar chart (imitation of Relevance Pooling method [[21]](https://paperpile.com/c/JWmey8/H7m0)).


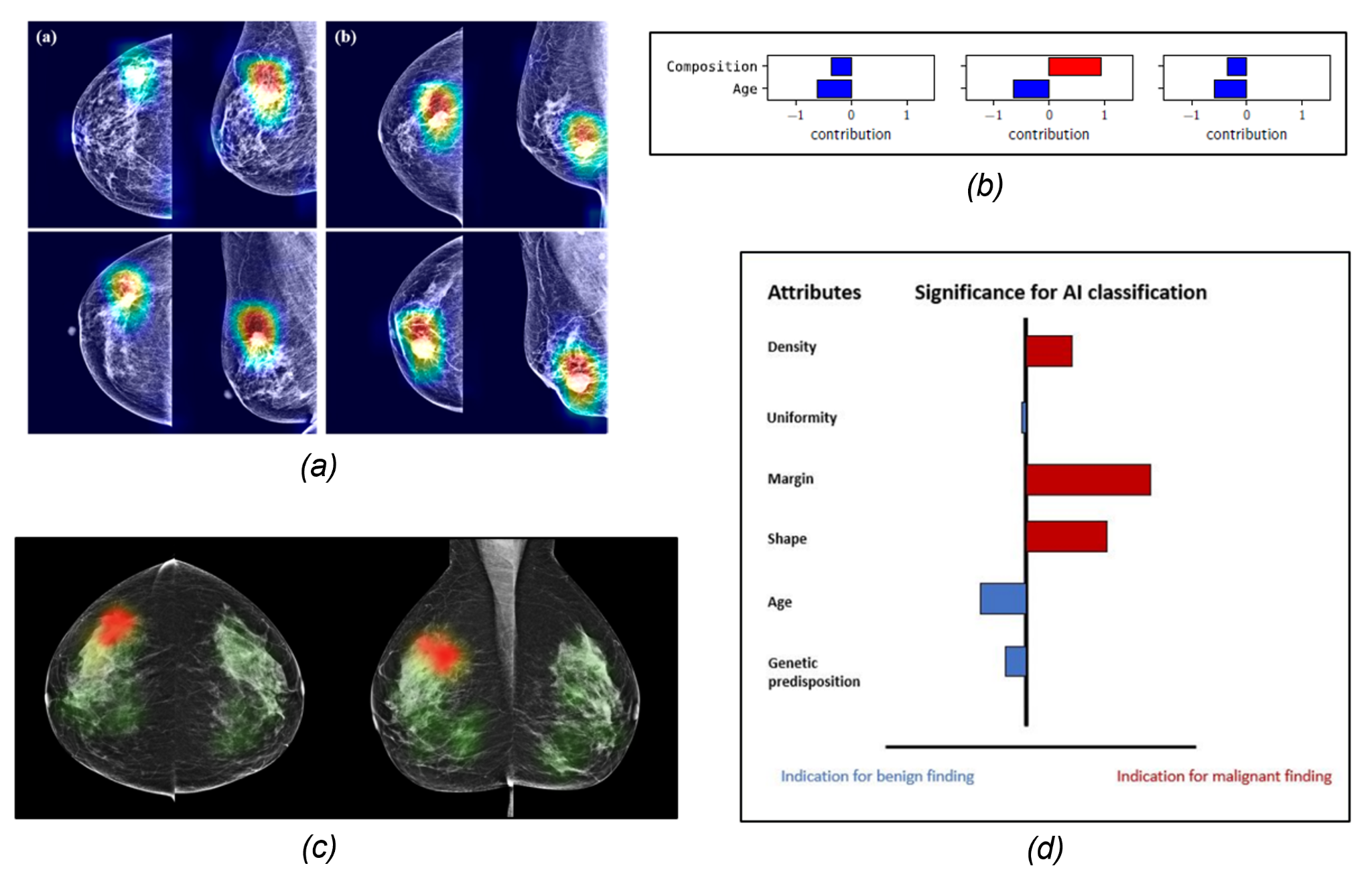


**Figure A3.** Distribution of participants across the control groups


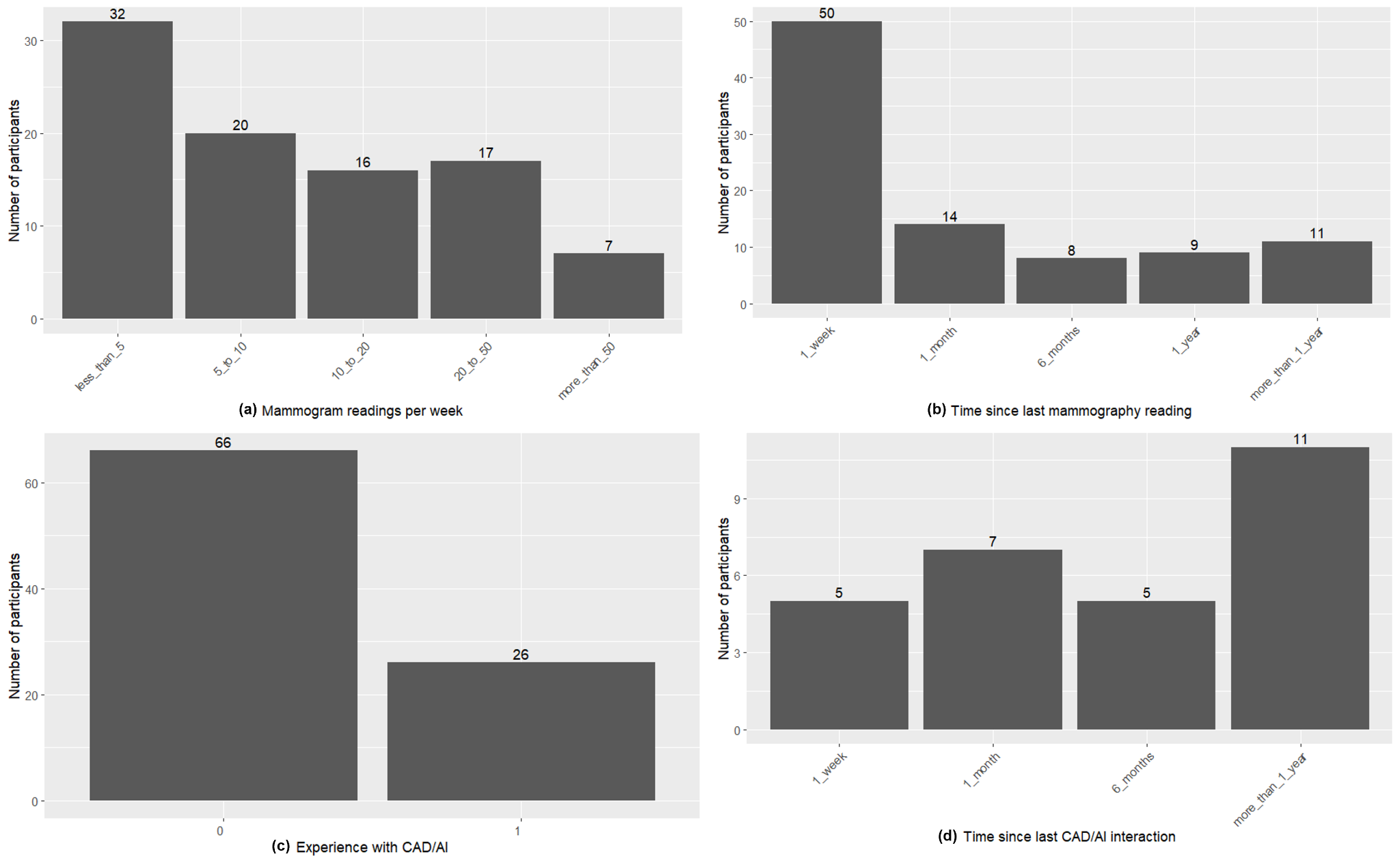


**Table A2.** Outcome variables

| **Concept** | **Measures / variables** |
| --- | --- |
| Decisions performance | - **Human Error** (Classification of participant - Ground Truth; *delta_part_true_l, delta_part_true_r*) - **Absolute Error** (Abs. value of Classification of participant - Ground Truth; abs(*delta_part_true_l)*, abs(*delta_part_true_r*) - **Deviation from AI suggestion** (Classification of participant - AI suggestion, *delta_part_ai*) |

**Table A3.** Control variables

| **Concept** | **Measures / variables** |
| --- | --- |
| Experience of performing the task | Overall experience in performing the task (mammography)  *control_nr_mamms_weekly* |
|  | Recent experience of performing tasks  *control_last_mamm* |
| Experience of using CAD/AI tools | Overall Experience with using CAD and/or AI tool  *control_cad_exp*  *control_ai_exp* |
|  | Recent experience with CAD and/or AI  *control_exp_last* |

**Table A4.** Descriptive statistics between the participants grouped after the control variable “*Time since last mammography reading”* in relation to the outcome variable *Human Error* (Human classification - Ground Truth)

|  | 1 Week | 1 Month | 6 Months | 1 Year | More than 1 Year | p-value |
| --- | --- | --- | --- | --- | --- | --- |
| Mean of human error (SD) | 0.05 (0,81) | 0 (0,79) | 0.17 (0,99) | 0.03 (0,75) | 0.05 (0,93) | 0.167 |

**Table A5.** Descriptive statistics between the participants grouped after the control variable “*Mammogram readings per week”* in relation to the outcome variable *Human Error* (Human classification - Ground Truth)

|  | Less than 5 | 5 to 10 | 10 to 20 | 20 to 50 | More than 50 | p-value |
| --- | --- | --- | --- | --- | --- | --- |
| Mean of human error (SD) | 0.03 (0,85) | 0.05 (0,81) | 0.06 (0,87) | 0.11 (0,79) | 0 (0,82) | 0.412 |

**Table A6.** Descriptive statistics between the participants grouped after the control variable “*Time since last CAD/AI interaction”* in relation to the outcome variable *Human Error* (Human classification - Ground Truth)

|  | 1 Week | 1 Month | 6 Months | More than 1 Year | p-value |
| --- | --- | --- | --- | --- | --- |
| Mean of human error | -0.01 (0,76) | 0.11 (0,89) | 0.03 (0,90) | 0.1 (0,85) | 0.503 |

**Figure A4.** Histogram of *time spent per case* (side-level, n = 2738; data was cleaned, 22 outliers above 500 seconds were not considered)


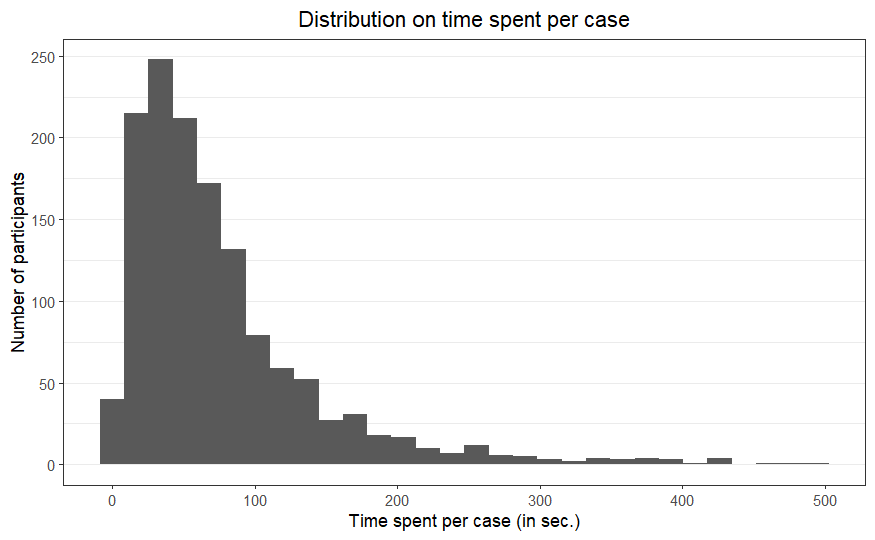


**Figure A5.** Plot of Human Error between cases where participants spent different amount of time (side-level, n = 2738; data was cleaned, 22 outliers above 500 seconds were not considered)


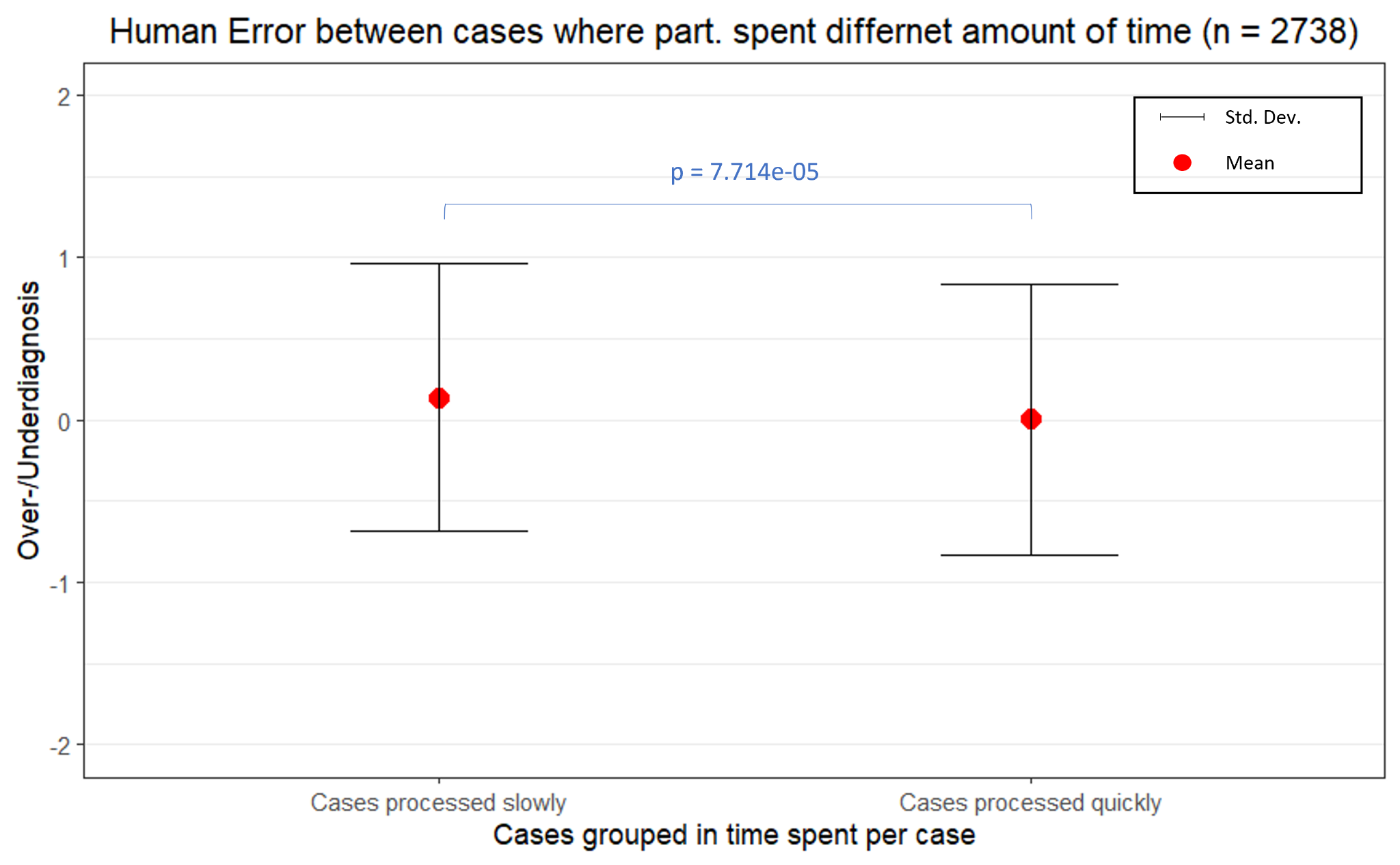


**Figure A6.** Classification time per case per participant group


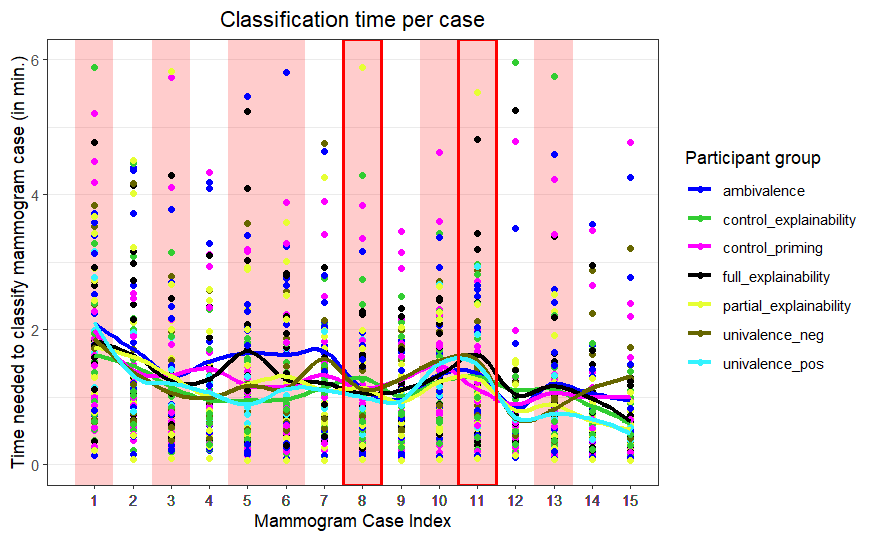


**Figure A7.** Histogram on how fast participants opened the AI suggestion per case relative to the case time

*
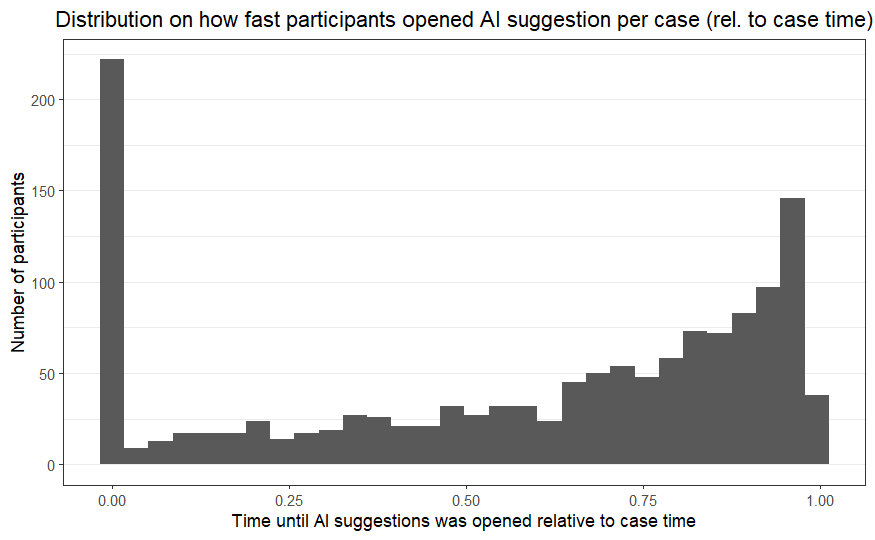
*

**Figure A8.** Histogram on how fast participants opened the Heatmap per case relative to the case time


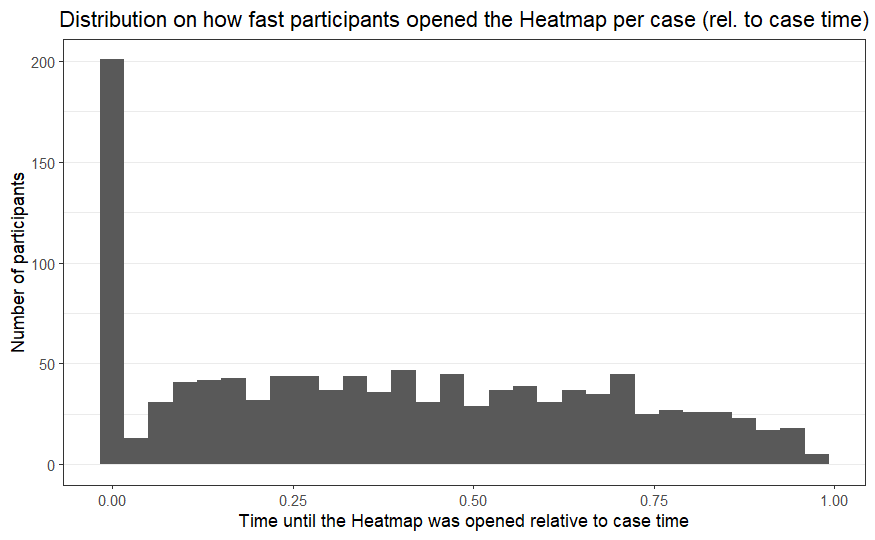


**Figure A9.** Histogram on how long participants opened the Heatmap per case relative to the case time


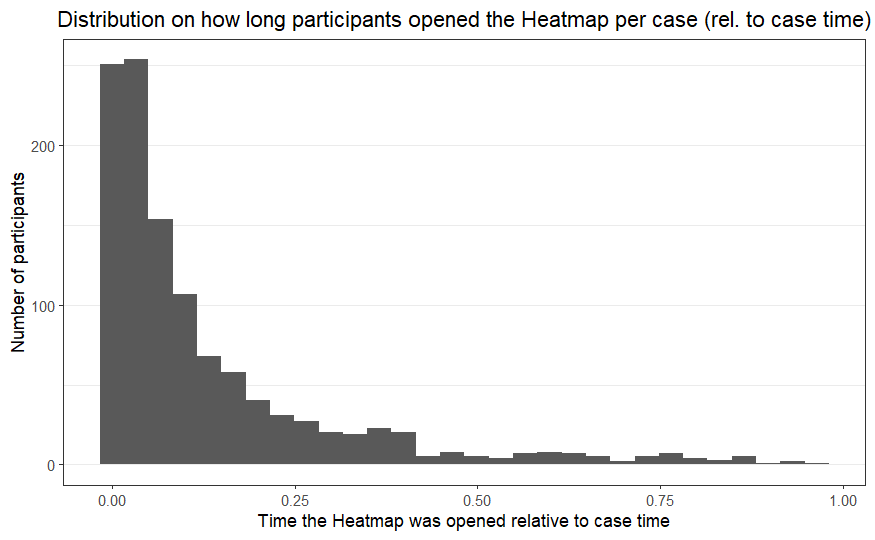


**Figure A10.** Sankey diagram of Diagnosis Outcome

**
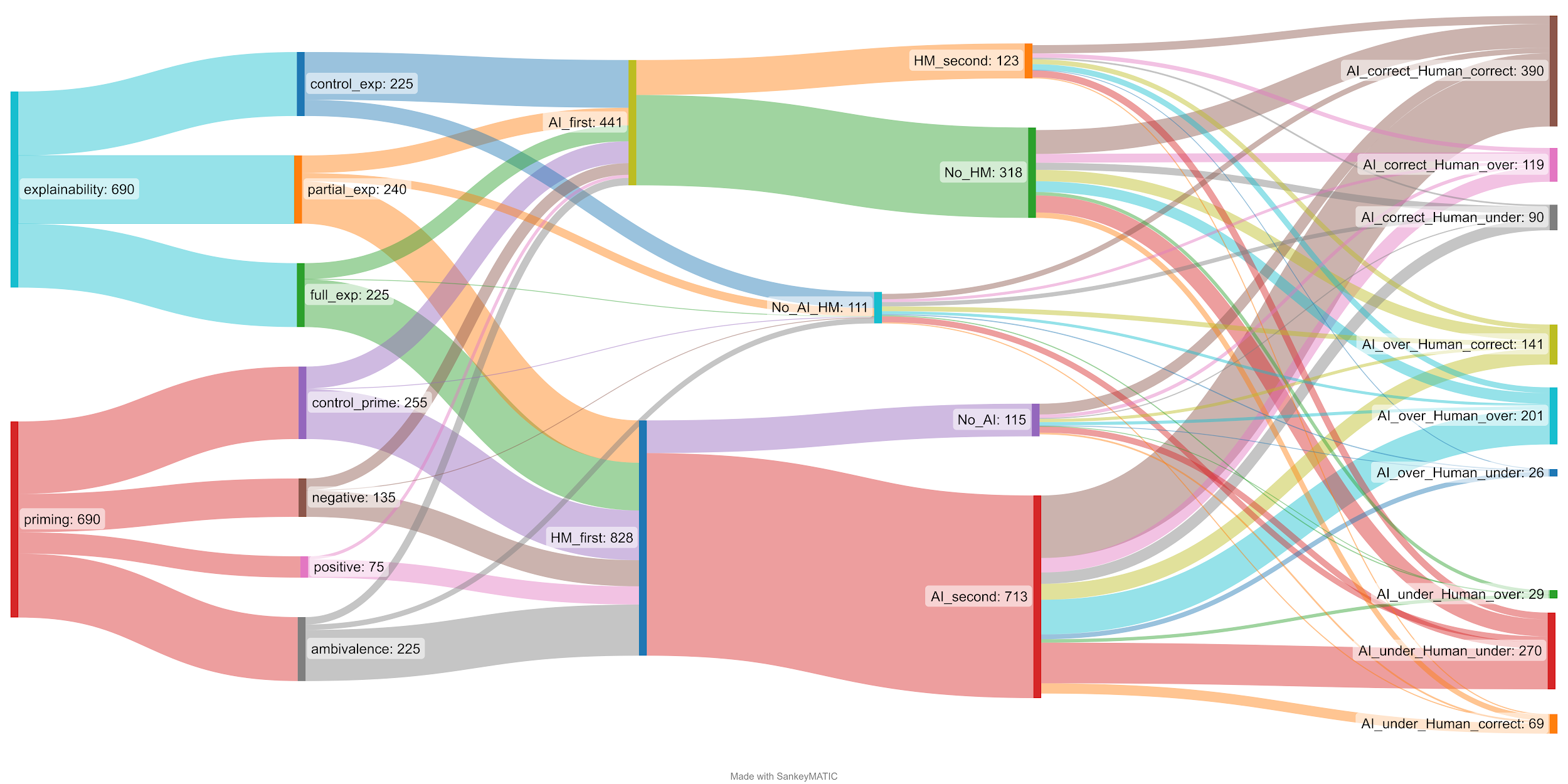
**

**Figure A11.** Plot of Human Error between Explainability groups (n = 1380)


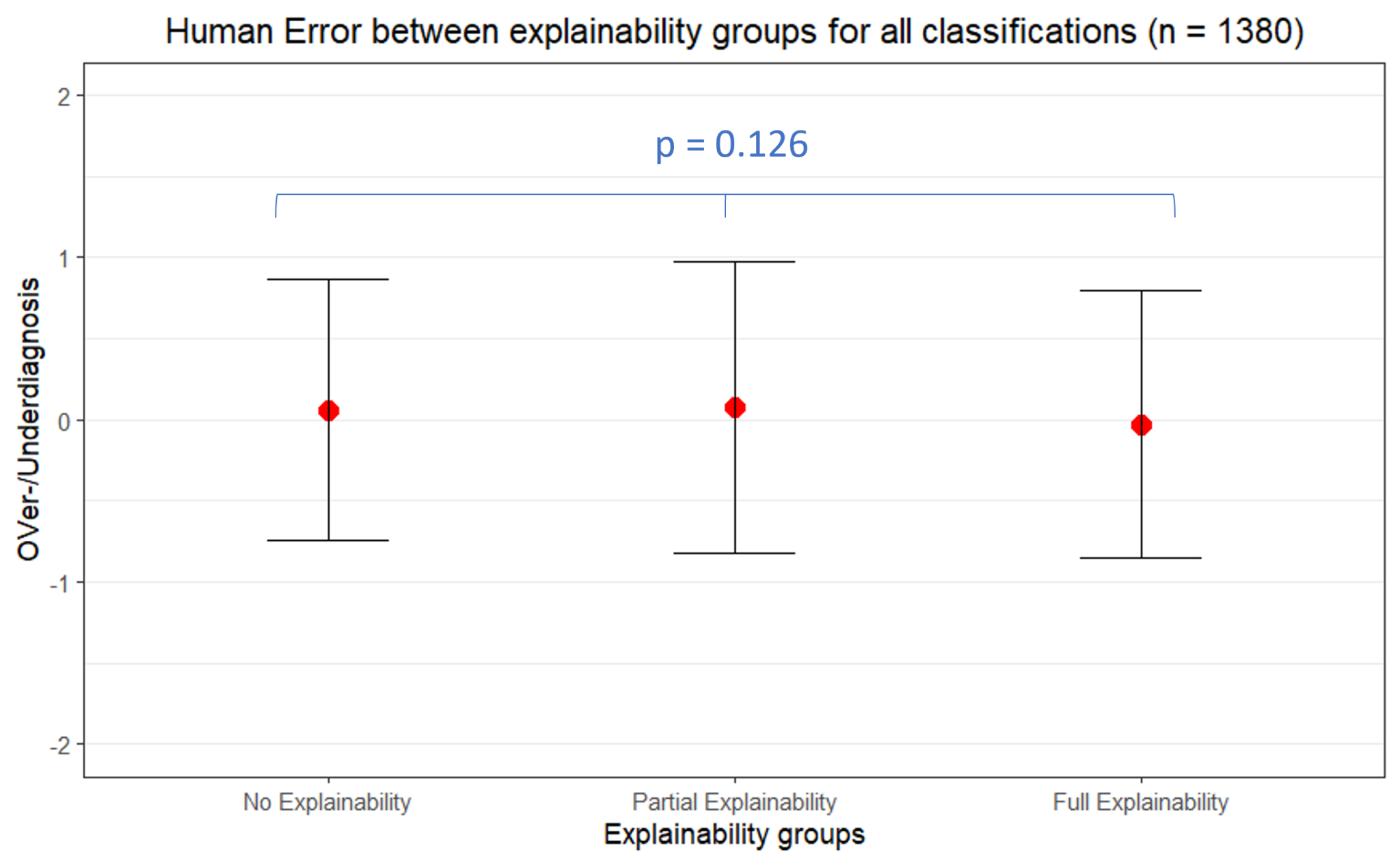


**Figure A12.** Plot of Human Error between Explainability groups (n = 1380)


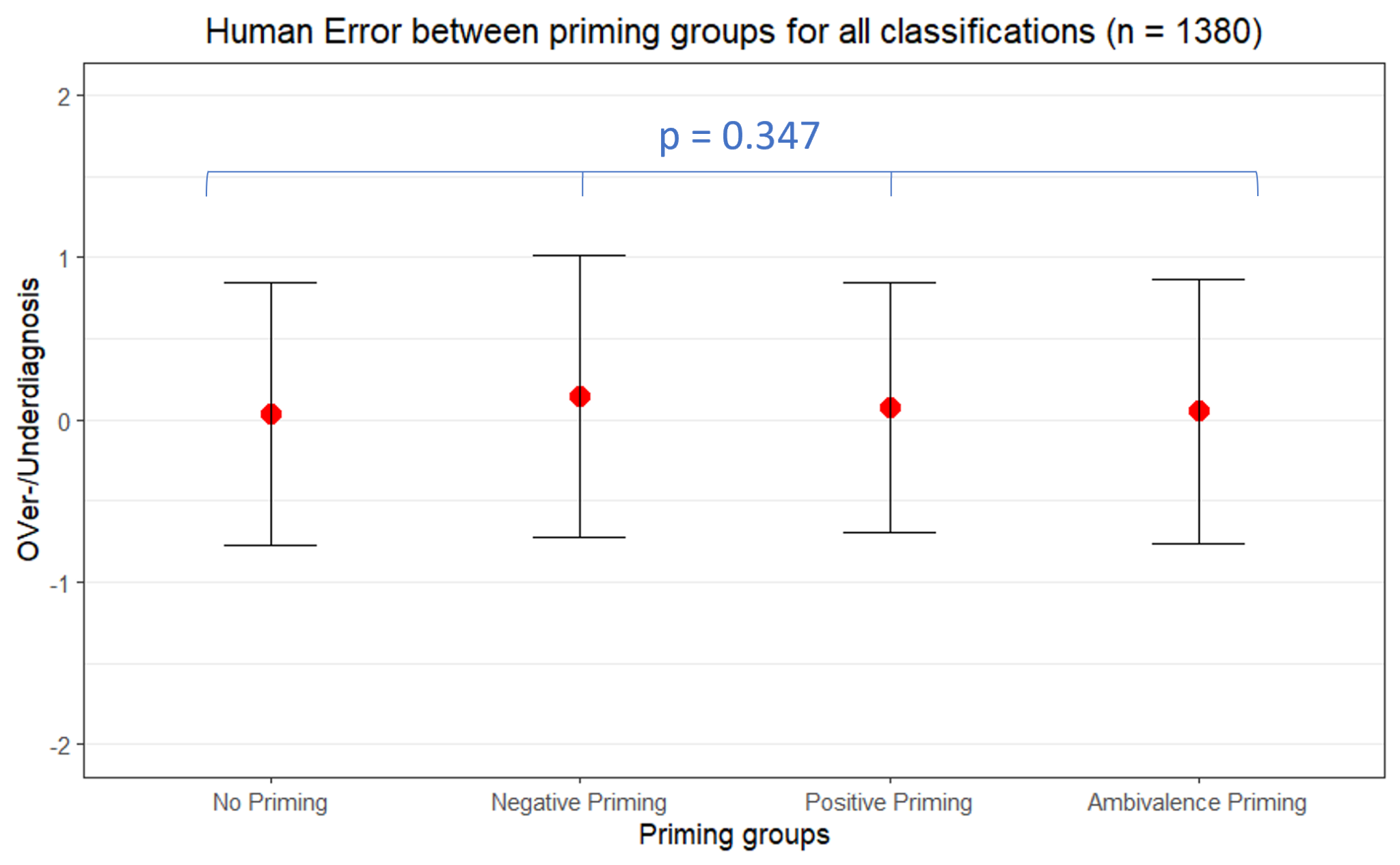


**Table A7.** Summary of pathways to correct, over- and underdiagnosis decisions

| **Diagnosis decision type** | **Pathways** | **Additional observations** |
| --- | --- | --- |
| **Correct diagnosis (64%)** | | |
| (1782/2760) 64% | Corr-1: Consulting correct suggestions (76%) | There is no pattern regarding the average time spent on the task, the frequency and recency of working with mammography, or experience with using CAD/AI tools. |
|  | Corr-2: Consulting incorrect suggestions (13%) | 57% spend less than average time  No difference in terms of frequency and recency of experience  Unlikely when positively primed (4%) |
|  | Corr-3: Not consulting suggestions (15%) | No such a pattern regarding the time spent  53% have recent experience (last month) and 56% had on average 5 mammogram per week |
| **Overdiagnosis (20%)** | | |
| Human Error = 1 or 2 | Over-1: Consulting overdiagnosis suggestions 33% | 62% spend below average time on the task  True scores are “2” (82%) or “3” (18%)  More likely to be in negative (22) than positive priming (15)  No specific pattern regarding the frequency and fresh experience (slightly less frequent than the average) |
|  | Over-2: Consulting correct suggestions (46%) | In 87%, the true score is “2” (tendency to avoid deeming cases normal!)  No difference in terms of recency and frequency of experience |
|  | Over-3: Consulting underdiagnosis suggestions (5%) | Mostly (17/27) spend below the average time  Only happens in cases “6” and “13” where AI deems both sides as normal (triggering them to find something in the images)  No difference in terms of the recent and frequency of experience and no specific pattern regarding the experimental groups |
|  | Over-4: Not consulting suggestions (16%) | Mostly (75%) spend less than average time  Happens in all cases! And slightly more frequently experienced than the average (78% of them have frequent experience with mammography at least 5 cases per week)  Only happens in cases with true score of “2” and “3” |
| Human Error = 3 | Over-5 (14/2760) 0,6% | No specific pattern,  Often when AI suggestion was correct (9 out of 14)  Mostly (10 out of 14) happened in cases “4” and “11” |
| **Underdiagnosis (16%)** | | |
| Human error = -1 or -2 | Under-1: Consulting underdiagnosis suggestions (53%) | No difference in terms of the time spent on the tasks, observed in all experimental groups (no specific pattern); no difference in terms of frequency of doing mammography nor their fresh experience. |
|  | Under-2: Consulting correct suggestions (24%) | In 58% are when the true score is “4” or “5”; no case of “2”  In 66%, they spent less than average time on the task  In 96% when they were not positively primed  No pattern regarding the recent experiences with the task  92% of them had frequent experience of doing mammography at least 5 cases per week |
|  | Under-3: Consulting overdiagnosis suggestions (4%) | Only on task “10-Left”  No specific pattern regarding experience or time spent on the task |
|  | Under-4: Not consulting AI suggestions (18%) | No specific pattern in terms of time spent on the case,  No specific pattern in terms of experience recency and frequency  Different prevalence across the groups |
| Human error = -3 | Under-5 (0,2%) | In all cases, AI offers correct suggestion and the true value is “5” |
