# Supplementary material for: The impact of AI suggestions on radiologists’ decisions: a pilot study of explainability and attitudinal priming interventions in mammography examination
Source: Sci Rep. 2023 Jun 7;13:9230. doi: 10.1038/s41598-023-36435-3 (PMC10247804; doi:10.1038/s41598-023-36435-3)
Supplement: Supplementary file 2 — Supplementary Information 2. [file 41598_2023_36435_MOESM2_ESM.docx]

Appendix B - Screens Experiment UI


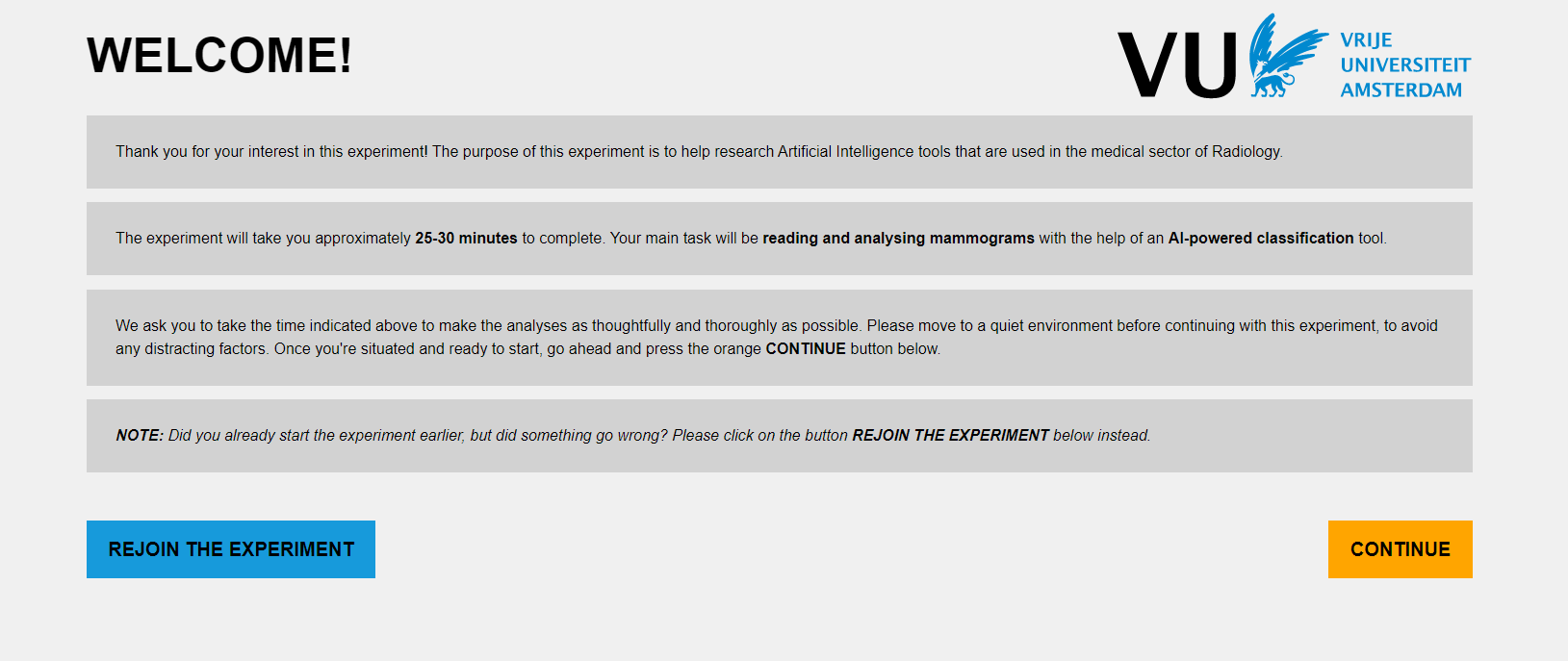


**Figure B1.** Home page of online experiment


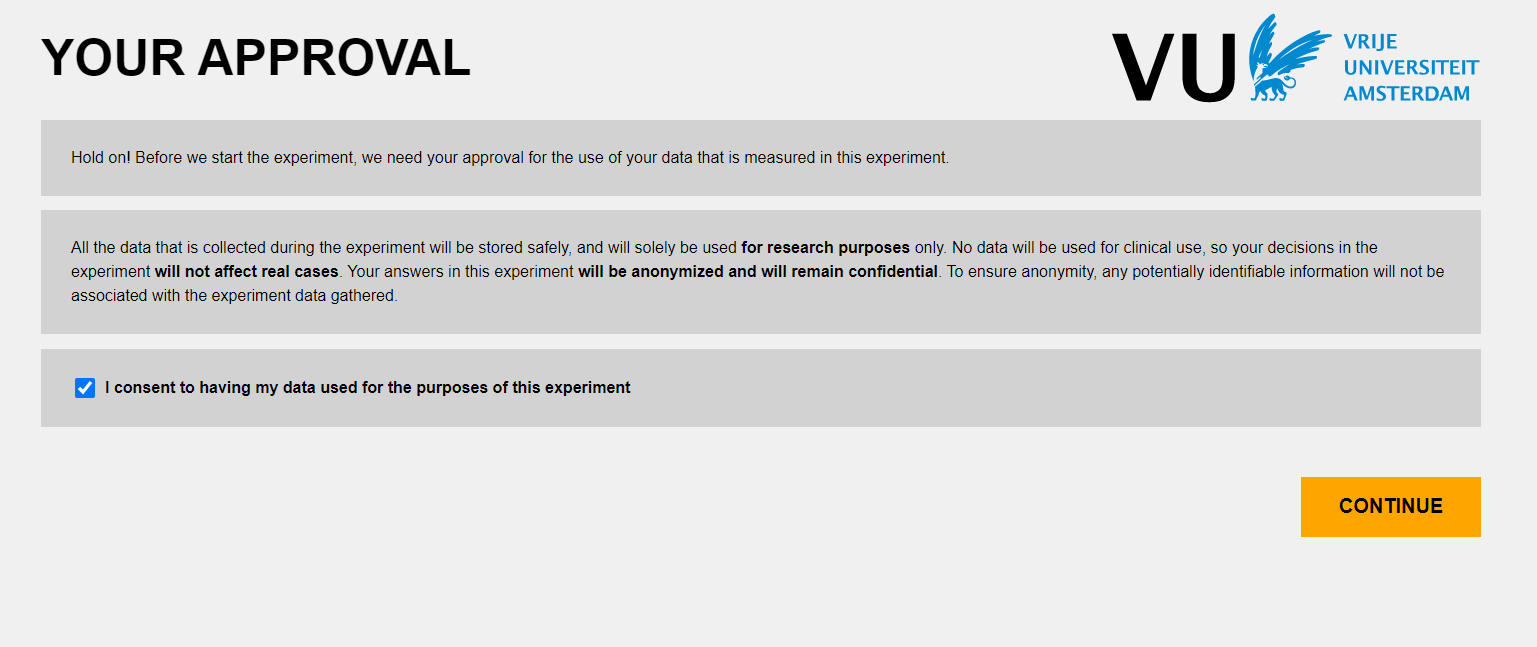


**Figure B2.** Consent form page of online experiment


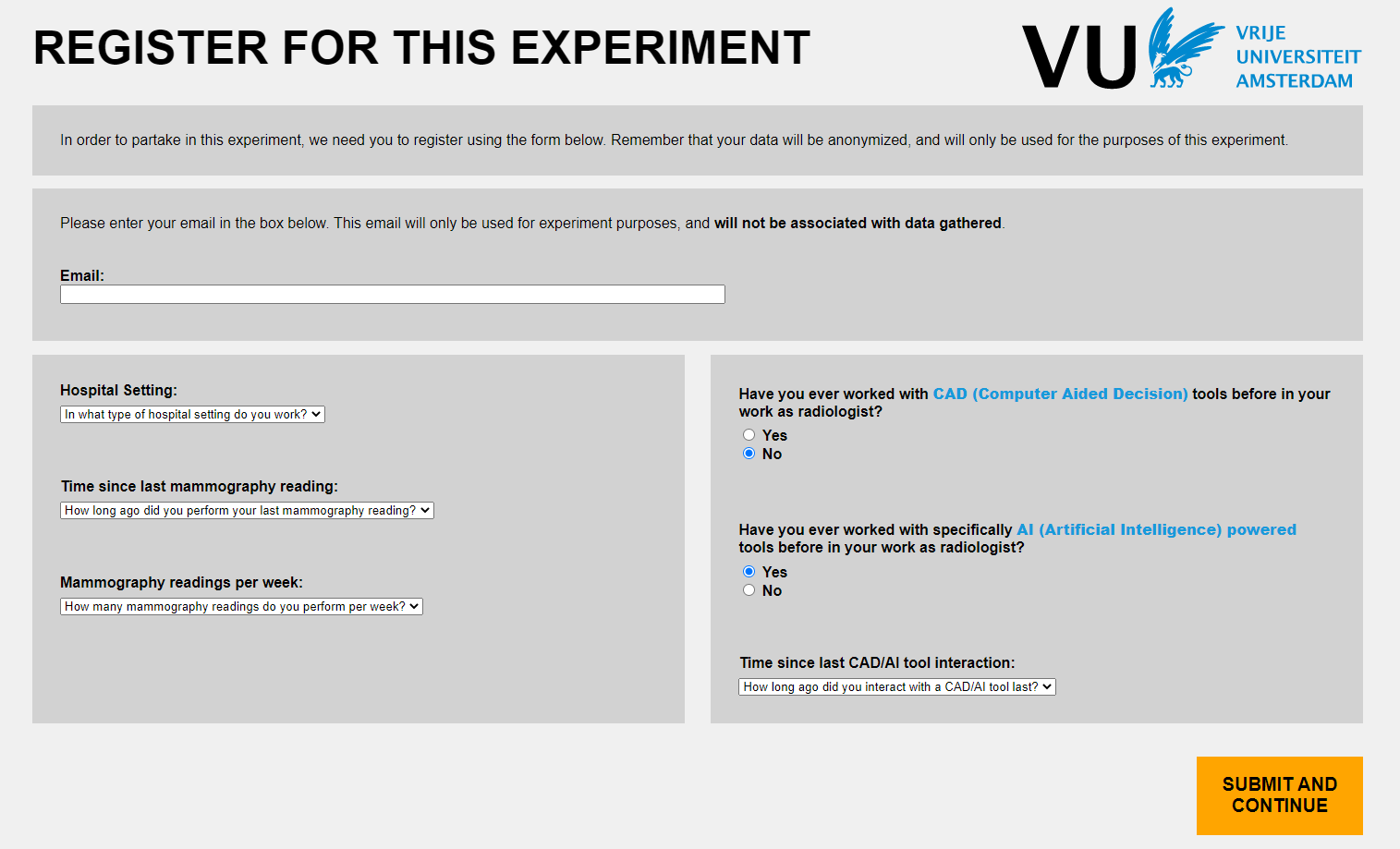


**Figure B3.** Registration page of online experiment


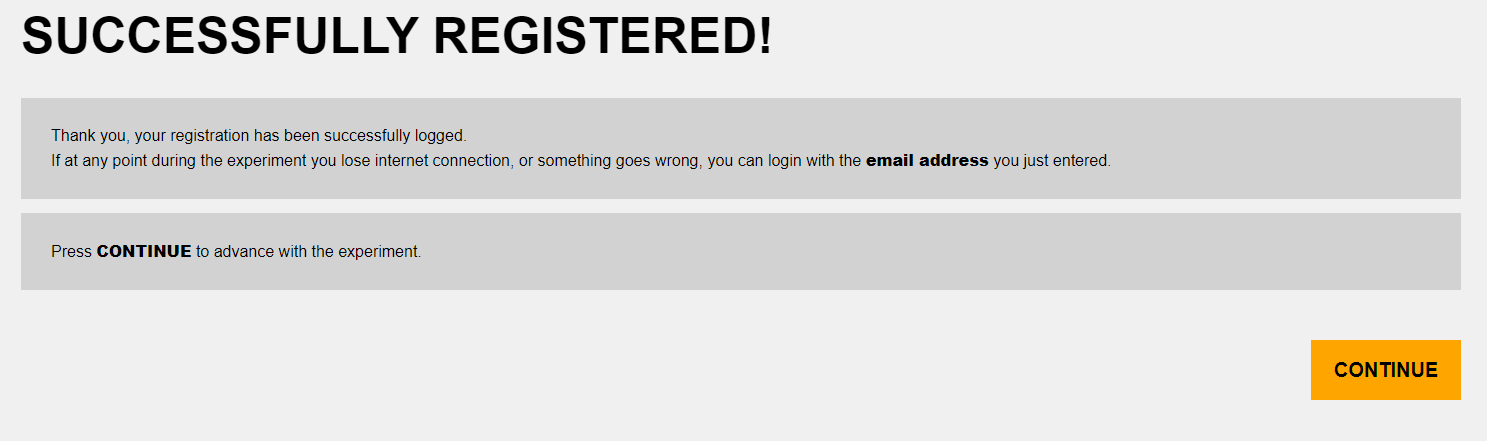


**Figure B4.** Registration success page of online experiment


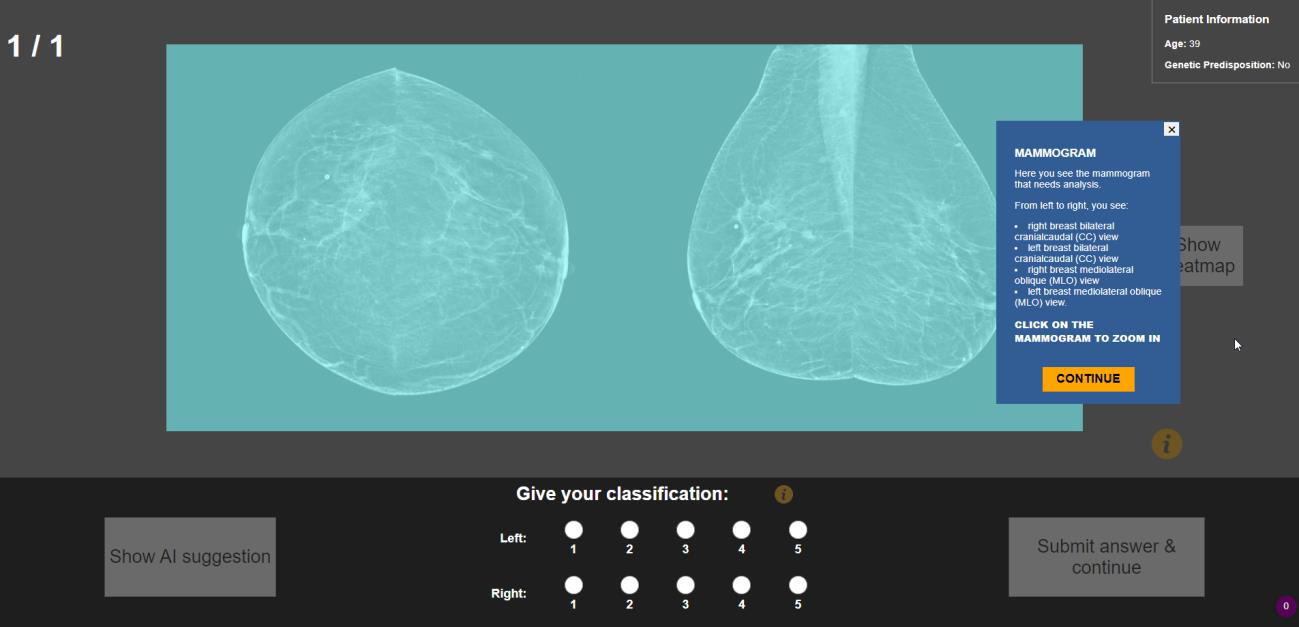


**Figure B5.** Interface tour page of online experiment


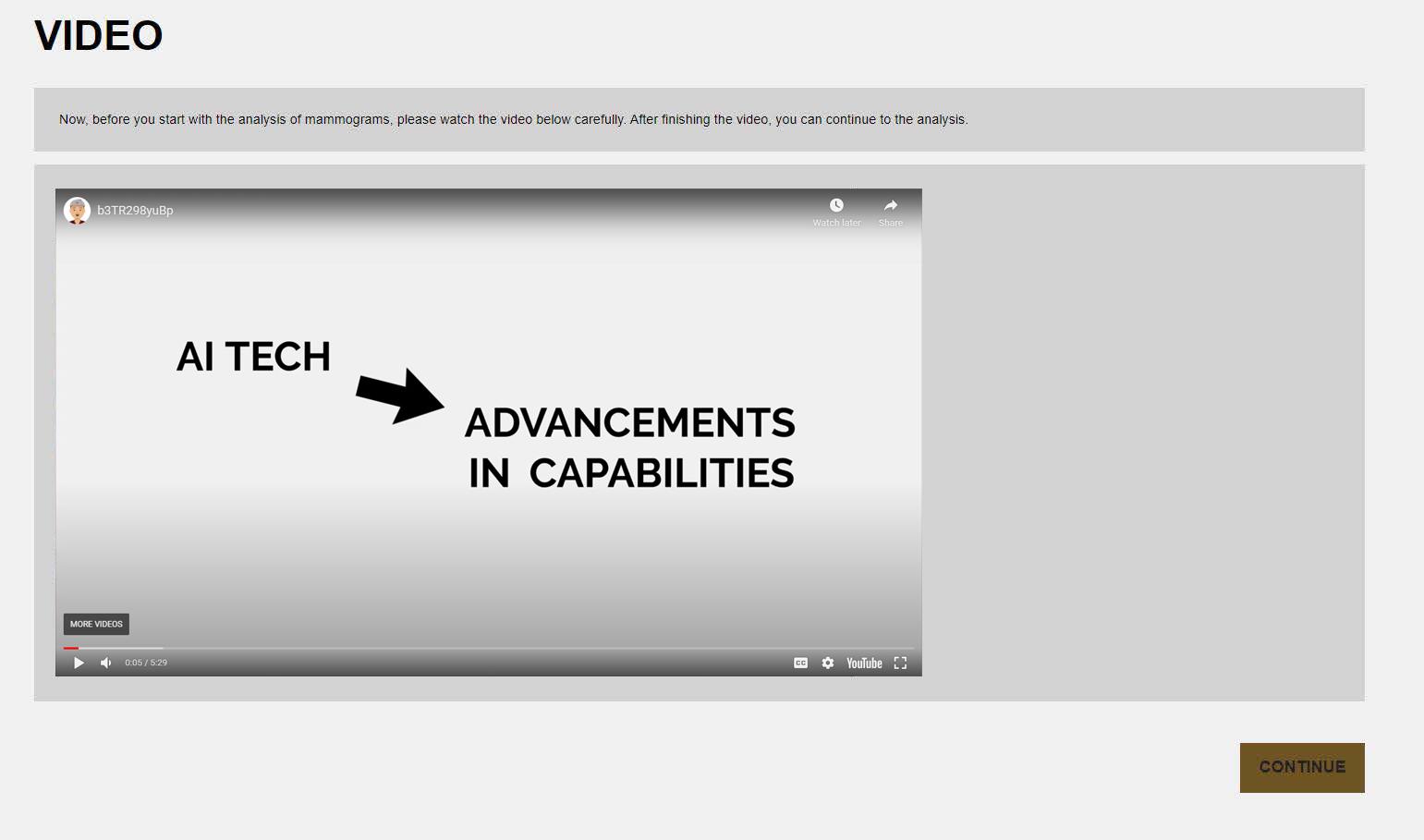


**Figure B6.** Priming video page of online experiment


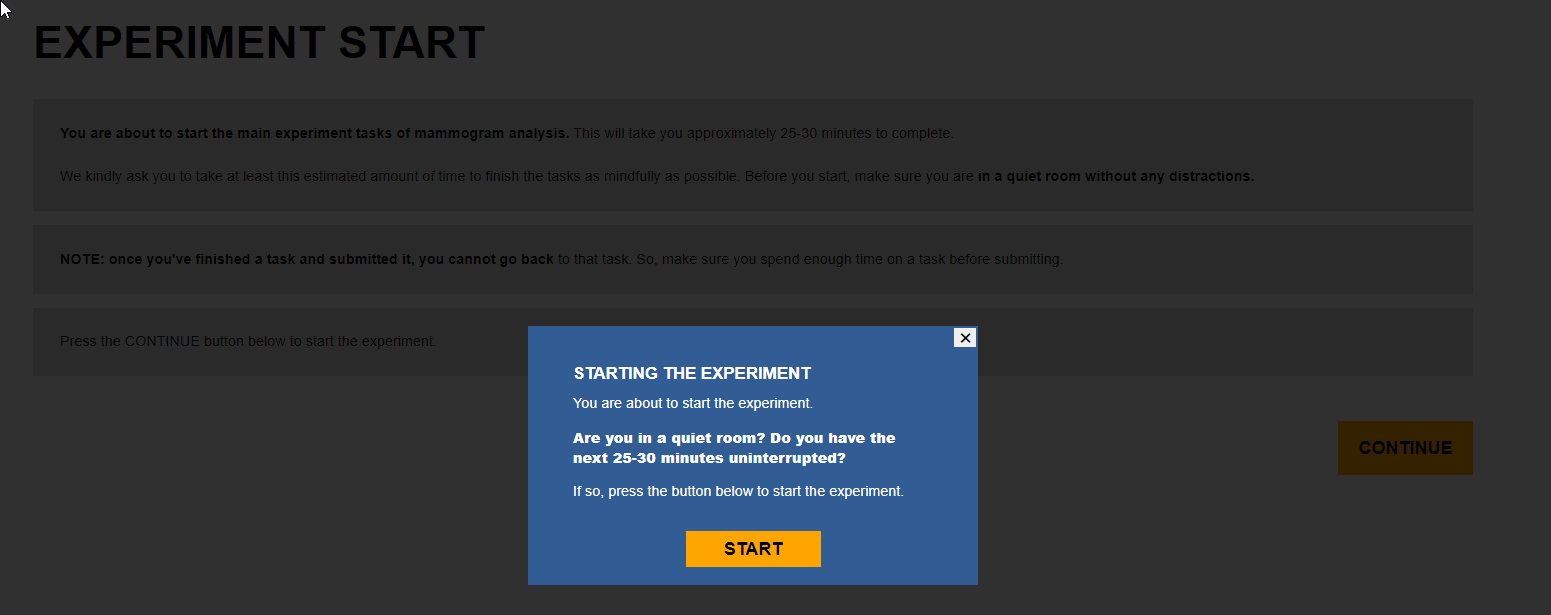


**Figure B7.** Pre-experimental task page of online experiment


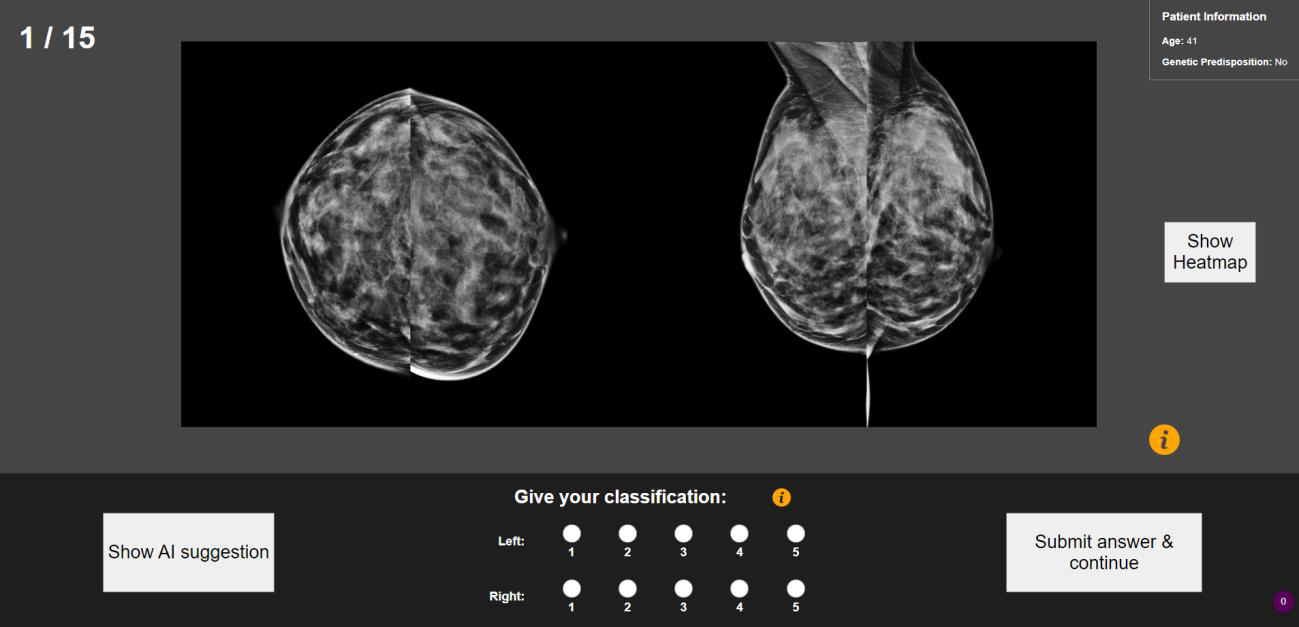


**Figure B8.** Experimental task page of online experiment


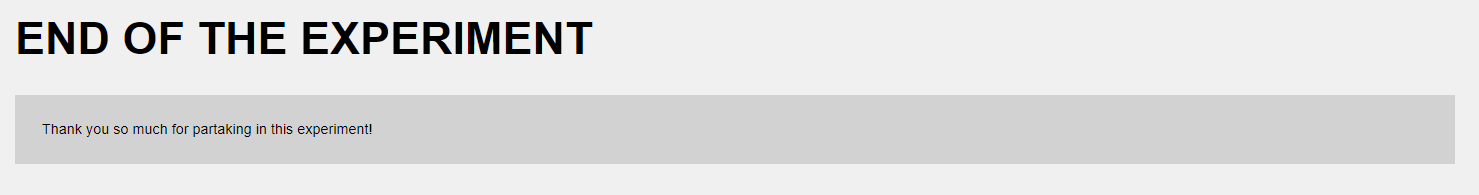


**Figure B9.** Final page of online experiment
