# Supplementary material for: The impact of AI suggestions on radiologists’ decisions: a pilot study of explainability and attitudinal priming interventions in mammography examination
Source: Sci Rep. 2023 Jun 7;13:9230. doi: 10.1038/s41598-023-36435-3 (PMC10247804; doi:10.1038/s41598-023-36435-3)
Supplement: Supplementary file 3 — Supplementary Information 3. [file 41598_2023_36435_MOESM3_ESM.docx]

Appendix C - Priming Videos & Sources

**Table C1.** URLs of the priming videos.

| **Priming Valence** | **Video URL** |
| --- | --- |
| ambivalence | <https://youtu.be/6So7SXo_-8A> |
| positive | <https://youtu.be/hdOcoHNEPKc> |
| negative | <https://youtu.be/iAoFAQcj990> |

**Table C2.** Sources and experts used in the development of the priming videos.

| **Experts in Video** | **Video Title/Source** |
| --- | --- |
| Prof. Enrico Coeira  *Founder of Australian Alliance for AI in Healthcare* | Will AI mean we no longer need doctors?  *TEDx - Macquarie University, Sydney Australia* |
| Dr. Jeanne Shen  *Associate Director, Center for AI in Medical Imaging*  Dr. Nigam H. Shaw  *Co-director, Center for AI in Medical Imaging*  Dr. Matthew P. Lungren  *Principal Clinical AI/ML, AWS* | The state of artificial intelligence in medicine  *Stanford Medicine - Stanford, California, USA* |
| Dr. Eric Topol  *Founder of Scripps Research Translational Institute* | Various videos on AI by TDC Group  *TDC Group - Napa, California, USA* |
| Prof. Joe Simmons  *Professor of Operations, Information, and Decisions* | Overcoming “Algorithm Aversion”  *Wharton School of University of Pennsylvania - Philadelphia, USA* |
