# Supplementary material for: The impact of AI suggestions on radiologists’ decisions: a pilot study of explainability and attitudinal priming interventions in mammography examination
Source: Sci Rep. 2023 Jun 7;13:9230. doi: 10.1038/s41598-023-36435-3 (PMC10247804; doi:10.1038/s41598-023-36435-3)
Supplement: Supplementary file 4 — Supplementary Information 4. [file 41598_2023_36435_MOESM4_ESM.docx]

<https://drive.google.com/drive/folders/1htOZ5FySqZPjuOejQ8cq335CO0PXT53f?usp=share_link>
