# Supplementary material for: The impact of AI suggestions on radiologists’ decisions: a pilot study of explainability and attitudinal priming interventions in mammography examination
Source: Sci Rep. 2023 Jun 7;13:9230. doi: 10.1038/s41598-023-36435-3 (PMC10247804; doi:10.1038/s41598-023-36435-3)
Supplement: Supplementary file 5 — Supplementary Information 5. [file 41598_2023_36435_MOESM5_ESM.docx]

**Definition of Data frame Columns**

**Table E1.** Variables measured in the study and their descriptions, in reference to the dataframe file

| **Column Name** | **Description** |
| --- | --- |
| participant_id | Unique participant ID |
| participant_type | ‘ambivalence’, ‘univalence’, or ‘control’ for participants of category type “priming”    ‘full_explainability’, ‘partial_explainability’, or ‘control’ for participants of category type “explainability” |
| category_type | Experiment type, ‘priming’ for Ferdi’s participants, ‘explainability’ for Marcel’s participants |
| video_bool_is_pos | If a participant is of type ‘univalence’, this variable indicates negatively valent (0) and positively valent (1). If not of type ‘univalence’, value NULL. |
| control_hospital | Control question: in what type of hospital setting do you work? |
| control_last_mamm | Control question: how long ago did you perform your last mammography reading? |
| control_nr_mamms_weekly | Control question: how many mammography readings do you perform per week? |
| control_cad_exp | Control question: have you ever worked with CAD (computer aided decision) tools before? |
| control_ai_exp | Control question: have you ever worked with specifically AI tools before? |
| control_exp_last | Control question: last time since working with a CAD or AI tool |
| total_training_time | Time spent on interface tour page (in ms) |
| task_id | ID number of the task (1-15) |
| birads_classification | The BI-RADS score analysis given by a participant to the mammogram presented in the experimental task, in JSON format.   - li = left mammogram score - re = right mammogram score |
| total_time_ai_prediction | Time spent until a participant accesses the AI BI-RADS suggestion (in ms). |
| total_time_open_heatmap | Time spent until a participant accesses the AI heatmap (in ms). This is created as a separate variable from total_time_ai_prediction, as participants were found to use the AI heatmap more than the AI suggestion. |
| total_time_prob_distr | A sum of all time spent inspecting the AI malignancy score information during an experimental task (in ms).  NULL if element was not presented in UI. |
| total_visits_pro _distr | A sum of how many times the AI malignancy score information was visited during an experimental task.  NULL if element was not presented in UI. |
| total_time_heatmap | A sum of all time spent inspecting the AI heatmap during an experimental task (in ms). |
| total_visits_heatmap | A sum of how many times the AI heatmap was opened during an experimental task. |
| total_time_contr_attr | A sum of all time spent inspecting the AI pooling bars information depicting contributing attributes during an experimental task (in ms).  NA if element was not presented in UI. |
| total_visits_contr_attr | A sum of how many times the AI pooling bars information was visited during an experimental task.  NULL if element was not presented in UI. |
| total_time_first_birads_class | Time spent until a participant enters their first BI-RADS values (in ms). |
| total_birads_class_changes | A sum of how many times a participant has changed their selected BI-RADS values before submitting an experimental task. This was included to account for possible decision changes. |
| total_time_class_submit | A sum of all time spent to submit an experimental task. |
| true_classification | The true BI-RADS analysis of an experimental task. |
| ai_classification | The BI-RADS analysis given by the AI to the mammogram presented in an experimental task. |
| abnormality_score | “AI generated” abnormality score, expressing probability percentage of malignant tissue. |
| correct_ai | Boolean, indicating if the value for **ai_classification** is the same (1) as **true_classification** or not (0). |
| genetic_predis | Boolean, indicating if the patient of the task has a genetic predisposition for malignant tissue growth. |
| age | Age of the patient of the experimental task. |
| task_order | Order of the task performed (from 1 – 15) |
| part_class_l | Birads value for the Left breast, given by participant. (li part of **birads_classification**)  **Note:** BIRADS value 1 is changed to 2 |
| part_class_r | Birads value for the right breast, given by participant. (re part of **birads_classification**)  **Note:** BIRADS value 1 is changed to 2 |
| true_class_l | True birads value for the Left breast. (li part of **true_classification**) |
| true_class_r | True birads value for the right breast. (re part of **true_classification**) |
| ai_class_l | Birads value for the Left breast, given by AI. (li part of **ai_classification**) |
| ai_class_r | Birads value for the right breast, given by AI. (re part of **ai_classification**) |
| delta_part_true | Difference between **birads_classification** and **true_classification**, counting classifications containing BIRADS value 1 as 2 |
| delta_part_ai | Difference between **birads_classification** and **ai_classification**, counting classifications containing BIRADS value 1 as 2 |
| delta_true_ai | Difference between **true_classification** and **ai_classification** |
| missclass | *misclassification* == √(delta_part_true)^2^ > 0 |
| ai_error_size | Indicates by how much an ai_classification deviates from true_classifcation (in BIRADS values): big_od / big_ud if delta = 2, small_od / small_ud if delta = 1. |
| ai_never_opened | Boolean, indicating whether the participant has opened the AI advice. If 0, this means the participant did not interact with the AI values at all. |
| heatmap_never_opened | Boolean, indicating whether the participant has opened the heatmap. If 0, this means the participant did not interact with the Heatmap at all. |
| total_time_tasks | Total time a participant has spent on the experimental tasks. (in seconds) |
| total_time_experiment | Total time a participant has spent on the full experiment (starting from the moment of registration, including explanation pages, priming, interface training, experimental tasks, and post-experiment questions) (in seconds) |

| ai_left_mistake | Boolean, indicates if AI made mistake on left (Li) BI-RADS class (1) or not (0) |
| --- | --- |
| ai_right_mistake | Boolean, indicates if AI made mistake on right (Re) BI-RADS class (1) or not (0) |
| analytical_interaction_heatmap | *(Analytical_interaction_heatmap = total_visits_heatmap IF total_visits_heatmap > 1)* *OR (Analytical_interaction_heatmap = 1 IF total_visits_heatmap == 1 AND total_time_heatmap < 0.9 * total_time_class_submit); is NA if total_time_class_submit is NA for respective case* |
| analytical_interaction_rpbc | *Analytical_interaction_rpbc = total_visits_contr_attr IF total_time_contr_attr > 3 sec.* |
| analytical_interaction_agg | Analytical_interaction_heatmap + Analytical_interaction_rpbc;  *is only* Analytical_interaction_rpbc *if* Analytical_interaction_heatmap *is NA for respective case* |
| heatmap_opened_before_AI | Boolean, indicates whether Heatmap was opened before AI prediction was opened (1) or AI prediction was opened before Heatmap was opened (0) |
| delta_part_true_l | delta_part_true → left side |
| delta_part_true_r | delta_part_true → right side |
| human_underdiagnosis_left | Human underdiagnosis for left mammogram, boolean |
| human_correct_diagnosis_left | Human correct diagnosis for left mammogram, boolean |
| human_overdiagnosis_left | Human overdiagnosis for left mammogram, boolean |
| human_underdiagnosis_right | Human underdiagnosis for right mammogram, boolean |
| human_correct_diagnosis_right | Human correct diagnosis for left mammogram, boolean |
| human_overdiagnosis_right | Human overdiagnosis for left mammogram, boolean |
| AI_underdiagnosis | AI underdiagnosis, boolean |
| AI_correct_diagnosis | AI correct diagnosis, boolean |
| AI_overdiagnosis | AI overdiagnosis, boolean |
| human_overdiagnosis | human_overdiagnosis_l + human_overdiagnosis_r |
| human_correct_diagnosis | human_correct_diagnosis_l + human_correct_diagnosis_r |
| human_underdiagnosis | human_underdiagnosis_l + human_underdiagnosis_r |
| time_to_ai | normalized value → *total_time_ai_prediction* / *total_time_class_submit* |
| time_to_first_class | normalized value → *total_time_first_birads_class* / *total_time_class_submit* |
| time_to_hm | normalized value → *total_time_open_heatmap* / *total_time_class_submit*  NA if *total_time_open_heatmap* = “NULL” or NA |
| tt_delta_ai_hm | absolute difference *total_time_ai_prediction* & *total_time_open_heatmap* |
| tt_delta_hm_fc | absolute difference *total_time_open_heatmap* & *total_time_first_birads_class* |
| tt_delta_ai_fc | absolute difference *total_time_ai_prediction* & *total_time_first_birads_class* |
| time_process | Variable that describes order of opening AI (ai), heatmap (hm), and giving first birads classification (fc)  e.g.: hm_fc_ai → first heatmap is opened, then first classification is given, then AI is checked.  Value is NA if:   - - - *total_time_class_submit* > 1000000     - *time_to_open_heatmap* = NA → participant_type = control_explainability     - *time_to_open_heatmap* = 0 → heatmap is never opened AND *time_to_open_ai* = 0 → AI suggestion is never opened |
| time_process_cc | Variable that captures **co-clicks (cc)**, so if AI (ai), heatmap (hm), or first classification (fc) are clicked within 2 seconds of each other  e.g.: cc_ai_hm → AI and heatmap are co-clicked  e.g.: cc_all → AI, heatmap, AND first classification are all co-clicked  Value is NA if no co-clicks are occurring for case.  This variable combines with variable **time_process** |
| time_hm | normalized value → *total_time_heatmap* / *total_time_class_submit*  NA if *total_time_heatmap* = “NULL” or NA |
| time_to_ai_classes | IF *time_to_ai* = NA -> NA  IF *time_to_ai* = 0 -> “ai_never_opened”  ELSE IF 0 < *time_to_ai* <= 0.33 -> “ai_opened_quickly”  ELSE IF 0.33 < *time_to_ai <= 0.66 ->* “ai_opened_moderatly_fast”  ELSE IF *time_to_ai > 0.66 ->* “ai_opened_late” |
| time_to_hm_classes | IF *time_to_hm* = NA -> NA  IF *time_to_hm* = 0 -> “hm_never_opened”  ELSE IF 0 < *time_to_hm* <= 0.33 -> “hm_opened_quickly”  ELSE IF 0.33 < *time_to_hm <= 0.66 ->* “hm_opened_moderatly_fast”  ELSE IF *time_to_hm > 0.66 ->* “hm_opened_late” |
| time_hm_classes | IF *time_hm* = NA -> NA  IF *time_hm* = 0 -> “No_watchtime_hm”  ELSE IF 0 < *time_hm* <= 0.33 -> “short_watchtime_hm”  ELSE IF 0.33 < *time_hm <= 0.66 ->* “medium_watchtime_hm”  ELSE IF *time_hm > 0.66 ->* “long_watchtime_hm” |
| control_last_mamm_num_linear | Categorical control variable values expressed in numeric values on linear scale:   - 1_week → 1 - 1_month → 2 - 6_months → 3 - 1_year → 4 - more_than_1_year → 5 |
| control_last_mamm_num_true | Categorical control variable values expressed in numeric values on true scale (weeks):   - 1_week → 1 - 1_month → 4 - 6_months → 24 - 1_year → 55 - more_than_1_year → 110 (estimate) |
| control_nr_mamms_weekly_num_linear | Categorical control variable values expressed in numeric values on linear scale:   - less_than_5 → 1 - 5_to_10 → 2 - 10_to_20 → 3 - 20_to_50 → 4 - more_than_50 → 5 |
| control_nr_mamms_weekly_num_true | Categorical control variable values expressed in numeric values on true scale (average nr of mamms):   - less_than_5 → 3 - 5_to_10 → 8 - 10_to_20 → 15 - 20_to_50 → 35 - more_than_50 → 60 (estimate) |
| sequence_first | Either AI, HM, or NA depending on which is opened first |
| sequence_second | Either AI, HM, or NA depending on which is opened second.  If sequence_first == ‘NA’, sequence_second will always be ‘NA’ |
| time_spent_per_case_classes | IF *total_time_class_submit* per case <= mean(*total_time_class_submit* of all cases) -> time_spent_per_case_classes = "case_processed_quickly"  ELSE time_spent_per_case_classes = "case_processed_slowly |
| side | Mammogram side analysed. ‘Left’ for the left mammogram, ‘right’ for the right mammogram. |
| AI_human_diagnosis_matrix_assign | Outcome space variable, discerning type of diagnosis for both AI and Human |
| pathway | Decisional pathway, as described in manuscript. |
| human_error | (human answer - correct answer): then it is a value between -3 (under diagnosis) and +3 (over diagnosis) |
| ai_error | AI score - True Score |
| human_deviation | Human score - AI score   - Means human is overdiagnosing compared with AI and - means Human is going to lower scores than AI |
